# Aldiminium Cations as Countercations to Discrete Main Group Fluoroanions

**DOI:** 10.3390/molecules28176270

**Published:** 2023-08-27

**Authors:** Evelin Gruden, Gašper Tavčar

**Affiliations:** Department of Inorganic Chemistry and Technology, Jožef Stefan Institute, Jamova 39, 1000 Ljubljana, Slovenia

**Keywords:** aldiminium-based salts, CAAC-precursors, fluorination, pentafluorosilicate, pentafluorogermanate, synthesis

## Abstract

The reactions of group 14 tetrafluorides (SiF_4_, GeF_4_, and SnF_4_) and group 15 pentafluorides (PF_5_, AsF_5_, and SbF_5_) with the CAAC-based trifluoride reagent [^Me^CAACH][F(HF)_2_] led to the isolation of salts containing discrete 5- or 6-coordinated fluoroanions. The syntheses of [^Me^CAACH][SiF_5_], [^Me^CAACH][GeF_5_], [^Me^CAACH][(THF)SnF_5_], and the structurally related [^Me^CAACH][(dioxane)SnF_5_], [^Me^CAACH][PF_6_], [^Me^CAACH][AsF_6_], and [^Me^CAACH][SbF_6_] are effective, selective and in high yield. All compounds were characterized by X-ray single-crystal structure analysis, NMR and Raman spectroscopy. It is worth noting that the synthesized [^Me^CAACH][GeF_5_] is a rare example of a structurally characterized compound with discrete [GeF_5_]^−^ anion, while [^Me^CAACH][(THF)SnF_5_] and [^Me^CAACH][(dioxane)SnF_5_] represent the first compounds with discrete octahedrally coordinated tin fluoride anions with incorporated solvent molecules. Finally, the aldiminium-based cation [^Me^CAACH]^+^ proved to be suitable for the stabilization of rare discrete main group fluoride anions.

## 1. Introduction

Cyclic (alkyl)(amino)carbenes (CAACs) are a class of compounds that have been extensively studied since their discovery in 2005 [1]. They have been shown to be among the most nucleophilic and electrophilic stable carbenes known to date [2]. Their electronic and steric properties make them suitable ligands for the stabilization of highly reactive and unusual main group and transition metal species in their low or high oxidation states [2,3,4]. CAACs can be prepared from the corresponding aldiminium-based salts by deprotonation. Nowadays, several methods for the preparation of CAAC precursors are known [2]. It all started with the discovery of aldiminium-based triflate salts by Bertrand’s group [1] and was extended with the synthesis of aldiminium-based chloride salts [5]. Both salts can be effectively deprotonated to form CAACs. Interestingly, CAAC complexes could also be prepared starting from CAAC(H)OH in aqueous solution [6]. Later, aldiminium-based tetrafluoroborate salts also found their use as CAAC precursors [7]. They are usually prepared by ion exchange from the corresponding chloride derivatives using KBF_4_ [8]. Recently, [^Me^CAACH][BF_4_] was prepared by Jana’s group from CAAC: carbene and NO[BF_4_] [9]. The ^Me^CAAC: carbene, which acted as a one-electron reducing agent, was chemically oxidized with NO[BF_4_] to form a transient radical cation, which was subsequently converted to [^Me^CAACH][BF_4_] by hydrogen abstraction [9]. The same method led to the formation of [^Me^CAACH][SbF_6_] when NO[SbF_6_] was used [9].

Aldiminium-based triflate and chloride salts can be used not only as CAAC precursors but also as unusual group transfer reagents in the chemistry of aluminum hydrides [10]. Recently, we have prepared a series of aldiminium-based fluoride and poly(hydrogen fluoride) compounds that can be used as nucleophilic fluorination reagents [11]. In particular, the aldiminium-based trifluoride salt [^Me^CAACH][F(HF)_2_] proved to be very effective in organic transformations due to its ability to convert benzyl bromides, 1- and 2-alkyl bromides, sulfonyls, and silanes to the target fluorides [11].

In this work, we tested the reactivity of the recently developed [^Me^CAACH][F(HF)_2_] with inorganic fluoride compounds, in particular with group 14 and 15 elements. Our main goal was to extend the knowledge of aldiminium-based compounds and to prepare a series of compounds that could potentially exhibit group transfer properties analogous to the corresponding triflate and chloride salts. It is well known that cations have a strong influence on the form of the anions. For example, it has been shown that the steric bulkiness of the [IPrH]^+^ cation is suitable for the stabilization of discrete [SiF_5_]^−^, and [GeF_5_]^−^ anions, while less sterically demanding cations preferentially form octahedrally coordinated [SiF_6_]^2−^ species [12]. With this in mind, we were particularly interested in whether the aldiminium-based reagent would allow the stabilization of discrete group 14 fluoroanions. 

## 2. Results and Discussion

### 2.1. Synthesis and Structural Characterization

In our work, we tested the reactivity of group 14 tetrafluorides (SiF_4_, GeF_4_, and SnF_4_) and group 15 pentafluorides (PF_5_, AsF_5_, and SbF_5_) using the CAAC-based trifluoride reagent [^Me^CAACH][F(HF)_2_]. Since the properties of the starting compounds differ significantly even within the same group, slightly different synthesis procedures were required to prepare salts with 5- or 6-coordinated fluoroanions. The synthesis and characterization of all salts are systematically presented in the following chapters. Crystallographic data for all newly characterized compounds are compiled in the Supporting Information.

#### 2.1.1. Group 14

**Scheme 1:**
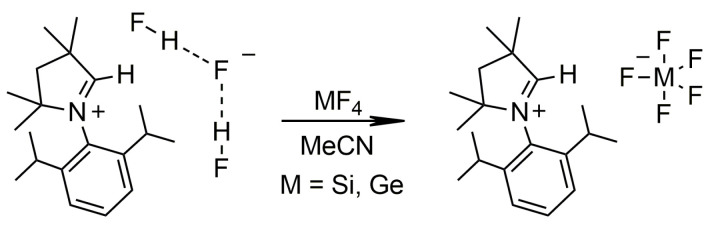
Synthesis of [^Me^CAACH][SiF_5_] and [^Me^CAACH][GeF_5_].

SiF_4_ and GeF_4_ in the gaseous state react with a solution of [^Me^CAACH][F(HF)_2_] in MeCN to form [^Me^CAACH][SiF_5_] or [^Me^CAACH][GeF_5_] in quantitative yield, as shown in Scheme 1. In the past, our group used a similar procedure to prepare the compounds with discrete anions—[IPrH][SiF_5_] and [IPrH][GeF_5_] [12]. Both newly prepared salts are stable at room temperature and can be stored under inert atmosphere for long periods of time.

**Figure 1:**
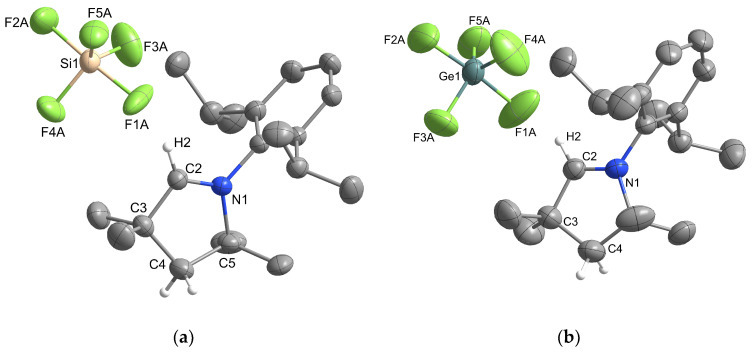
Structure of the asymmetric unit of (**a**) [^Me^CAACH][SiF_5_] and (**b**) [^Me^CAACH][GeF_5_]. The ellipsoids are drawn at 50% probability. The positions of the fluorine atoms in both anions are disordered. For clarity, only the fluorine atoms in domain A are shown and all hydrogen atoms except those on the aldiminium ring are omitted.

Single crystals of [^Me^CAACH][SiF_5_] and [^Me^CAACH][GeF_5_] were prepared by vapor diffusion crystallization using DCM as solvent and cyclopentane as antisolvent. The crystal structures of [^Me^CAACH][SiF_5_] and [^Me^CAACH][GeF_5_] are shown in Figure 1. Both salts crystallize in the monoclinic space group *P*2_1_/*n* and their asymmetric units consist of a heterocyclic cation [^Me^CAACH]^+^ and a discrete pentacoordinated anion. The [SiF_5_]^−^ and [GeF_5_]^−^ anions have a trigonal bipyramidal geometry with disordered positions of the fluorine atoms. Two preferred orientations were modeled and refined, with domain A and B occupancies in [SiF_5_]^−^ of 75% and 25%, respectively, and domain A and B occupancies in [GeF_5_]^−^ of 67% and 33%, respectively. The crystal structures with disordered anions are shown along with separate images of domains A and B in Figures S31 and S32 in the Supporting Information. The crystal structures of [SiF_5_]^−^ anions are often disordered and rarely well determined [13,14]. The structural features of our [SiF_5_]^−^ anion in [^Me^CAACH][SiF_5_] are consistent with the previously reported structures [15,16,17], but their bond lengths and angles are not compared with the previously reported structures due to the heavy disorder of the anion. The structural features of our [GeF_5_]^−^ anion in [^Me^CAACH][GeF_5_] are also consistent with the previously described [IPrH][GeF_5_] [12]. It is worth noting that [IPrH][GeF_5_] represents the first example in which the discrete [GeF_5_]^−^ anion is present, while [^Me^CAACH][GeF_5_] is only the second example. To stabilize discrete [SiF_5_]^−^ and [GeF_5_]^−^ anions, large cations must be used [12,17], and the sterically demanding cation [CAACH]^+^ was found to be suitable for this purpose.

**Scheme 2:**
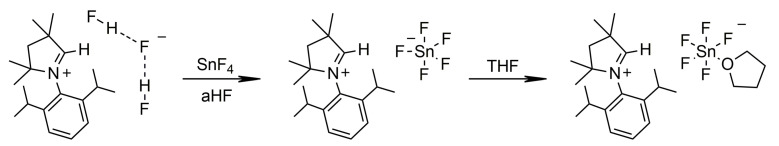
Synthesis of [^Me^CAACH][(THF)SnF_5_].

The SnF_4_ reacted with [^Me^CAACH][F(HF)_2_] in anhydrous HF with quantitative formation of [^Me^CAACH][SnF_5_], as shown in Scheme 2. Removal of the volatiles resulted in the formation of a brown solid that could not be crystallized despite several attempts. Several attempts have been made in the past to crystallize the monomeric [SnF_5_]^−^ anion, but none of them was successful [18]. Since tin preferentially forms octahedrally coordinated units in the presence of fluoride, [SnF_5_]^−^ units usually form oligomeric or polymeric structures [19]. Octahedrally coordinated discrete tin fluoride anions, such as [SnF_6_]^2−^, are more common and better studied [20,21,22]. However, only a limited number of structurally characterized [SnF_6_]^2−^ anions with organic cations are known. To date, there are only 12 discrete structures in the CCDC crystal structure database [23].

**Figure 2:**
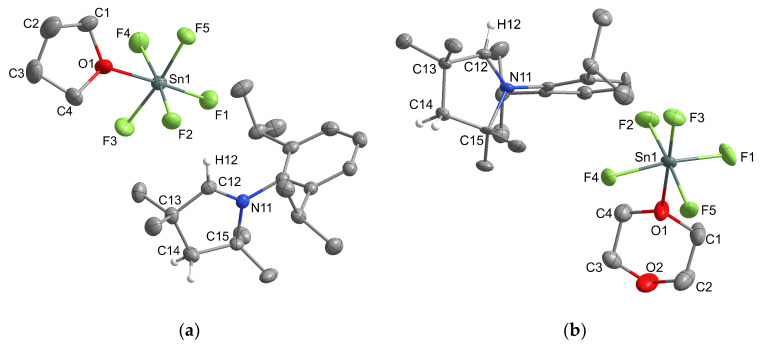
Crystal structure of (**a**) [^Me^CAACH][(THF)SnF_5_] and (**b**) [^Me^CAACH][(dioxane)SnF_5_]. The ellipsoids are drawn at 50% probability. For clarity, all hydrogen atoms except those on the heterocyclic ring are omitted.

In our work, we wanted to demonstrate the presence of [SnF_5_]^−^ units in a discrete form. Fortunately, [^Me^CAACH][SnF_5_] dissolved in THF and reacted with it to form a new compound. Immediately, a white solid precipitated from the THF solution, which was later characterized as a salt containing octahedrally coordinated [(THF)SnF_5_]^−^ anions. Single crystals of [^Me^CAACH][(THF)SnF_5_] suitable for X-ray analysis formed by vapor diffusion crystallization using THF as solvent and hexane as antisolvent. When [^Me^CAACH][SnF_5_] was suspended in dioxane, it reacted in the same way as with THF to form [^Me^CAACH][(dioxane)SnF_5_]. The latter compound was crystallized by vapor diffusion crystallization, using dioxane as solvent and hexane as antisolvent and formed single crystals of [^Me^CAACH][(dioxane)SnF_5_]∙dioxane. The crystal structures of the two compounds are shown in Figure 2.

[^Me^CAACH][(THF)SnF_5_] crystallizes in the monoclinic space group *C*2/*c*, while [^Me^CAACH][(dioxane)SnF_5_]∙dioxane crystallizes in the triclinic space group *P*−1. The asymmetric units of both compounds contain a heterocyclic cation [^Me^CAACH]^+^ and a discrete octahedrally coordinated anion. In addition, the asymmetric unit of [^Me^CAACH][(dioxane)SnF_5_]∙dioxane also contains two halves of the solvent dioxane (see Figure S33 in the Supporting Information). As far as we know, similar discrete structures with solvents have not been published before. So far, only the structurally related [RSnF_5_]^−^ anions (R = (Me_2_N)_2_C and C_5_H_10_N_2_) have been described by Röschenthaler’s group, with a carbene attached to the [SnF_5_]^−^ unit at the sixth coordination site [24]. Nevertheless, the structures of the [(THF)SnF_5_]^−^ and [(dioxane)SnF_5_]^−^ anions are consistent with the two structurally related species. The trans Sn–F distances of 1.945(2) Å and 1.941(2) Å for [(THF)SnF_5_]^−^ and [(dioxane)SnF_5_]^−^ anions, respectively, are slightly shorter but still in the range of the trans Sn–F distances of the related [RSnF_5_]^−^ anions (1.972(2) Å and 1.955(3) Å [24]). The same is true for the average cis Sn–F distances of 1.944 Å and 1.929 Å for [(THF)SnF_5_]^−^ and [(dioxane)SnF_5_]^−^ anions, respectively, which are shorter but still in agreement with the cis Sn–F distances of the related [RSnF_5_]^−^ anions (1.976 Å and 1.973 Å [24]). The difference from the literature data can be attributed to the higher electronegativity of the O-donor ligands, which strengthen the Sn–F bonds. The Sn–O bond distances of 2.167(2) Å and 2.177(2) Å for [(THF)SnF_5_]^−^ and [(dioxane)SnF_5_]^−^ anions, respectively, are also in agreement with the Sn–O distances of other structurally characterized compounds (2.134(2) Å [25], 2.1530(13) Å [26]).

#### 2.1.2. Group 15

**Scheme 3:**
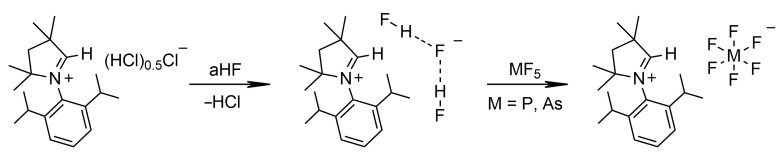
Synthesis of [^Me^CAACH][PF_6_] and [^Me^CAACH][AsF_6_].

**Scheme 4:**
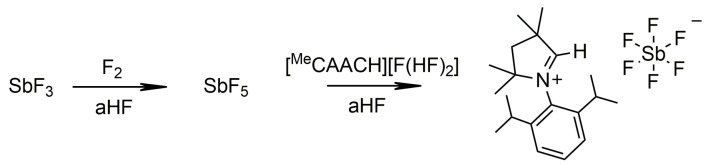
Synthesis of [^Me^CAACH][SbF_6_].

The one pot syntheses of [^Me^CAACH][PF_6_] and [^Me^CAACH][AsF_6_] are shown in Scheme 3. First, [^Me^CAACH][F(HF)_2_] was generated in situ by the reaction of [^Me^CAACH][Cl(HCl)_0.5_] in aHF. MF_5_ gases (M = P, As) were then added in excess to the reaction mixture, which was stirred overnight. After removal of volatiles, [^Me^CAACH][MF_6_] (M = P, As) was formed in quantitative yield. We were also able to prepare [^Me^CAACH][SbF_6_] using a similar procedure (Scheme 4). In this case, the SbF_5_ was first freshly prepared in situ from SbF_3_ and F_2_ in aHF. Then, [^Me^CAACH][F(HF)_2_] was added to the reaction mixture. To avoid the presence of chlorine atoms in the reaction mixture, [^Me^CAACH][F(HF)_2_] was used. Similar procedures have been used in the past for the synthesis of compounds containing [AsF_6_]^−^, and [SbF_6_]^−^ anions [27]. [^Me^CAACH][SbF_6_] was previously prepared and structurally characterized by Jana’s group [9]. Our results agree well with the reported data and provide a simple alternative approach for the formation of [^Me^CAACH][SbF_6_] in high yields. 

**Figure 3:**
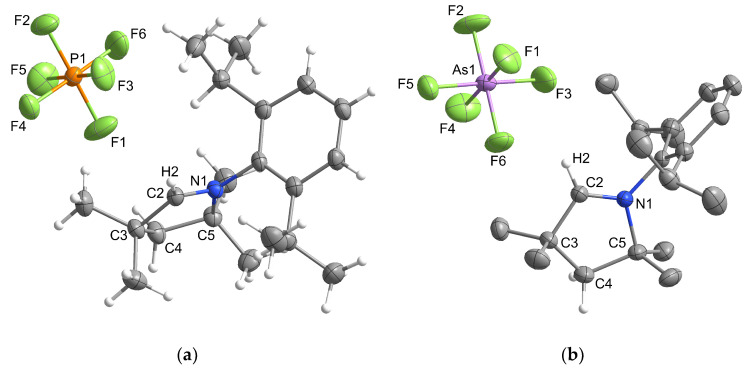
Structure of the asymmetric units of (**a**) [^Me^CAACH][PF_6_] and (**b**) [^Me^CAACH][AsF_6_]. The ellipsoids are drawn at 50% probability. For clarity, all hydrogen atoms except those on the heterocyclic ring are omitted.

Single crystals of all three compounds were prepared by vapor diffusion crystallization using DCM as solvent and cyclopentane as antisolvent. [^Me^CAACH][PF_6_] and [^Me^CAACH][AsF_6_] both crystallize in the orthorhombic space group *Pbca* and their crystal structures are shown in Figure 3. 

The two compounds are isostructural, with the hexafluoroarsenate compound having only a slightly larger unit cell. The asymmetric units of both compounds contain a heterocyclic cation [^Me^CAACH]^+^ and a discrete octahedrally coordinated anion [MF_6_]^–^ (M = P, As). The structural features of the anions agree well with the structures of the previously described species. The average P–F distance of 1.591 Å of [^Me^CAACH][PF_6_] is consistent with the average P–F distances of 1.570 Å [28], 1.588 Å [29], or 1.600 Å [29] reported elsewhere. Similarly, the slightly longer average As–F distance of 1.710 Å of [^Me^CAACH][AsF_6_] is comparable to the average As–F distances of 1.715 Å [30] and the range of 1.691(2)–1.757(2) Å reported for other [AsF_6_]^–^ anions [27].

#### 2.1.3. CAAC Precursors

**Scheme 5:**
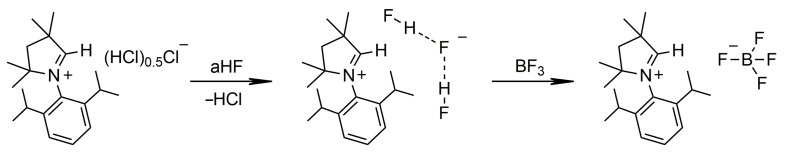
Synthesis of [^Me^CAACH][BF_4_].

Finally, we prepared the [^Me^CAACH][BF_4_] salt by the same procedure described for [^Me^CAACH][PF_6_] and [^Me^CAACH][AsF_6_]. The in situ prepared [^Me^CAACH][F(HF)_2_] reacted with BF_3_ gas and quantitatively formed [^Me^CAACH][BF_4_], as shown in Scheme 5. The same compound was recently prepared by Jana’s group from CAAC: carbene and NO[BF_4_] [9]. Our approach provides a simple and straightforward alternative for the preparation of CAAC-based tetrafluoroborate compounds in high yield. 

**Figure 4:**
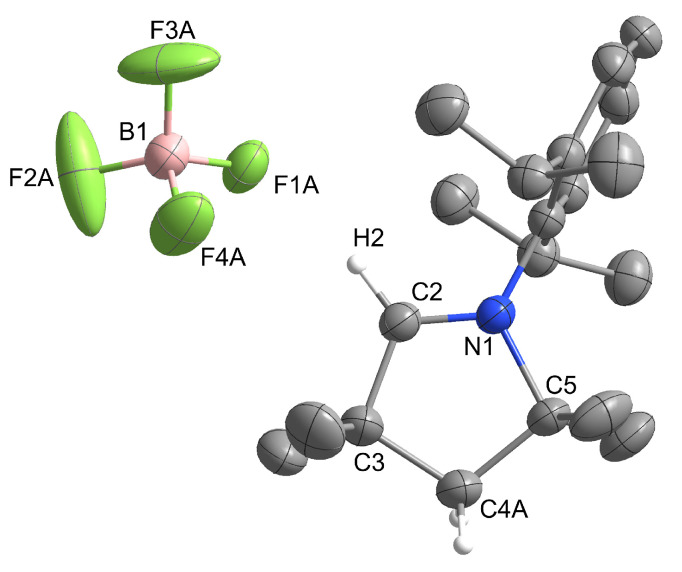
Structure of the asymmetric unit of [^Me^CAACH][BF_4_]. The ellipsoids are drawn at 50% probability. The positions of the fluorine atoms in the anion are disordered. For clarity, only the fluorine atoms in domain A are shown and all hydrogen atoms except those on the heterocyclic ring are omitted.

Single crystals of [^Me^CAACH][BF_4_] suitable for X-ray diffraction were prepared by vapor diffusion crystallization using DCM as solvent and cyclopentane as antisolvent. [^Me^CAACH][BF_4_] crystallizes in the monoclinic space group *P*2_1_/*n* and its crystal structure is shown in Figure 4. The asymmetric units contain a heterocyclic cation [^Me^CAACH]^+^ and a discrete, tetrahedrally coordinated [BF_4_]^–^ anion. The positions of the fluorine atoms in the anion are disordered. Two preferred orientations were modeled and refined, with domains A and B occupied by 73% and 27%, respectively. The crystal structures with disordered anions are shown along with separate images of domains A and B in Figure S34 in the Supporting Information. The structural features of our [BF_4_]^−^ anion in [^Me^CAACH][BF_4_] are consistent with the previously described structures [8], however, the bond lengths and angles are not compared to the previously reported structures due to the heavy disorder of the [BF_4_]^−^ anion.

**Figure 5:**
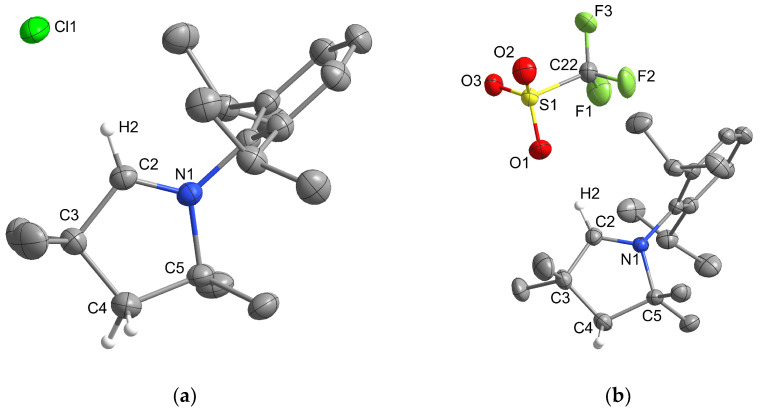
Crystal structures of (**a**) [^Me^CAACH][Cl] and (**b**) [^Me^CAACH][OTf]. The ellipsoids are drawn at 50% probability. For clarity, all hydrogen atoms except those on the heterocyclic ring are omitted.

In addition to the tetrafluoroborate salts, the CAAC-based chloride and triflate salts are still used as precursors for CAAC: carbenes. In the past, we have demonstrated their usefulness as group transfer reagents in aluminum hydride chemistry [10]. During our work, we succeeded in preparing [^Me^CAACH][Cl] and [^Me^CAACH][OTf] according to known procedures from the literature [1,10], crystallizing them and structurally characterizing them. The crystal structures of [^Me^CAACH][Cl] and [^Me^CAACH][OTf] are shown in Figure 5. [^Me^CAACH][Cl] crystallizes in the monoclinic space group *P*2_1_/*c*, while [^Me^CAACH][OTf] crystallizes in the monoclinic space group *P*2_1_/*n*. The asymmetric unit of [^Me^CAACH][Cl] contains a heterocyclic cation [^Me^CAACH]^+^ and a discrete chloride anion, while the asymmetric unit of [^Me^CAACH][OTf] contains two ion pairs consisting of a [^Me^CAACH]^+^ cation and an [OTf]^–^ anion. The asymmetric unit of [^Me^CAACH][OTf] is shown in Figure S35 in the Supporting Information. 

### 2.2. NMR Spectroscopy

The ^1^H, ^13^C, ^19^F, and other heteronuclear NMR spectra are given in the Supporting Information for all synthesized salts. All spectra are consistent with crystal structures determined by single-crystal X-ray diffraction. In the ^1^H and ^13^C NMR spectra, the characteristic signals for the [^Me^CAACH]^+^ cation are visible. In addition, the ^1^H and ^13^C NMR spectra of [^Me^CAACH][(THF)SnF_5_] contain signals for THF and residual hexane. 

Measured ^19^F and other heteronuclear NMR peaks (^11^B, ^29^Si, ^119^Sn, ^31^P, ^75^As, and ^121^Sb) are listed in Table 1. The ^74^Ge NMR spectra could not be measured due to the characteristics of the broadband probe. The frequency of ^74^Ge was outside the range of the broadband probe used. The data collected for the discrete fluoroanions of the main group elements agree well with the data reported for other related compounds. 

The ^19^F and ^11^B NMR signals of our [BF_4_]^–^ anion (^19^F: −151.94 ppm; ^11^B: −1.23 ppm) agree well with the data reported by Jana’s group for the same compound (^19^F: −151.49 ppm and −151.54 ppm; ^11^B: −1.19 ppm) [9]. 

Also, the ^19^F NMR signals of the [SiF_5_]^–^ and [GeF_5_]^–^ anions in [^Me^CAACH][SiF_5_] (^19^F: −137.53 ppm) and [^Me^CAACH][GeF_5_] (^19^F: −137.45 ppm) agree well with the previously characterized [SiF_5_]^–^ (^19^F: −139 ppm [13], −138.4 ppm [14], and −137.08 ppm [12]) and [GeF_5_]^–^ anions (^19^F: −136.60 ppm [12]). The ^29^Si NMR signal of [SiF_5_]^–^ in [^Me^CAACH][SiF_5_] (^29^Si: −149.86 ppm) is also consistent with the previously reported data for [SiF_5_]^–^ anions (^29^Si: −147.5 ppm [14]). 

Since the [(THF)SnF_5_]^–^ anion is not symmetrical, two distinct signals corresponding to the anion are seen in the ^19^F NMR spectra. A doublet is observed at −160.37 ppm belonging to the 4 cis-fluorine atoms and a quintet at −170.20 ppm belonging to the trans-fluorine atom. The same NMR pattern was observed by Röschenthaler’s group for [RSnF_5_]^−^ anions (R = (Me_2_N)_2_C and C_5_H_10_N_2_) [24]. The ^119^Sn NMR spectrum for the [(THF)SnF_5_]^–^ anion contains a multiplet at −789.93 ppm. Our results are in a similar range to those reported for [RSnF_5_]^−^ anions (^119^Sn: –749.75 ppm) [24]. The ^119^Sn NMR spectra of [RSnF_5_]^−^ anions give a doublet of quintets with ^1^*J*_SnF(cis)_ = 2160 Hz and ^1^*J*_SnF(trans)_ = 1490 Hz [24]. The ^119^Sn NMR spectrum of our [(THF)SnF_5_]^–^ anion has a similar shape with _1_*J*_119SnF_ = 1880 Hz. However, not all peaks are visible in the NMR spectrum due to the high background noise. 

The ^19^F and heteronuclear NMR spectra of the discrete fluoroanions of group 15 were much better resolved. The ^19^F NMR signal of the [PF_6_]^–^ anion in [^Me^CAACH][PF_6_] (^19^F: −72.84 ppm with *J* = 706.7 Hz) is a doublet due to coupling with ^31^P (*I* = 1/2), while its ^31^P NMR signal (^31^P: −146.09 ppm with *J* = 707.1 Hz) has the form of a septet due to coupling with ^19^F (*I* = 1/2). The values are in good agreement with previously characterized [PF_6_]^–^ anions (^19^F: −70.7 ppm with *J* = 704 Hz [31]; −70.2 ppm with *J* = 711.4 Hz [28]; ^31^P: −144.2 ppm with *J* = 711.3 Hz [28]). 

The ^19^F NMR signal of the [AsF_6_]^–^ anion in [^Me^CAACH][AsF_6_] (^19^F: −65.77 ppm with *J* = 931.2 Hz) is split into four lines due to scalar coupling with ^75^As (*I* = 3/2), while its ^75^As NMR signal (^75^As: 5.45 ppm with *J* = 931.6 Hz) is in the form of a septet due to coupling with ^19^F (*I* = 1/2). Our results are consistent with previously published data for [AsF_6_]^–^ anions (^19^F: −68.4 ppm [31]; –56.8 ppm [32];, –56.1 ppm [32]; ^75^As: 0.0 ppm [32]).

The ^19^F NMR signal of the [SbF_6_]^–^ anion in [^Me^CAACH][SbF_6_] consists of two sets of peaks. The first set of peaks with slightly higher intensity (^19^F: −123.96 ppm with *J* = 1936.3 Hz) is split into six lines due to scalar coupling with ^121^Sb (*I* = 5/2, natural abundance 57.25%), while the second set of peaks with slightly lower intensity (^19^F: −123.96 ppm with *J* = 1050.4 Hz) is split into eight lines due to scalar coupling with ^123^Sb (*I* = 7/2, natural abundance 42.75%). The chemical shift of the ^19^F NMR spectra agrees well with the previously reported results for [SbF_6_]^–^ anions (^19^F: −113.69 ppm [31]). The difference of about 10 ppm can be attributed to the use of a different NMR solvent. 

### 2.3. Raman Spectroscopy

The spectra of all compounds prepared can be found in Figures S28–S30 in the Supporting Information, while selected Raman peaks for M–F vibrations are listed in Table 2. Assignment of M–F vibrations was difficult due to the strong vibration of the [^Me^CAACH]^+^ cation in the same region, so only the strongest M–F vibrations were assigned. In the case of [^Me^CAACH][BF_4_], assignment of the B–F bands was not possible. The vibrations typical of [BF_4_]^−^ anions are normally found around 777 cm^−1^ [33]. However, in our case, it is very likely that the peaks are hidden under the peaks of the [^Me^CAACH]^+^ cation. The Si–F and Ge–F vibrations of the [SiF_5_]^−^ and [GeF_5_]^−^ anions were assigned to 712 cm^−1^ and 665 cm^−1^, respectively, which is consistent with the previously reported data for [IPrH][SiF_5_] and [IPrH][GeF_5_] (709 cm^−1^ and 664 cm^−1^, respectively) [12]. The Sn–F vibrations in [^Me^CAACH][(THF)SnF_5_] were assigned to 581 cm^−1^, which is in agreement with the reported Sn–F vibrations in octahedrally coordinated [SnF_6_]^2−^ anions (600 cm^−1^ [19], 592 cm^−1^ [33]). The P–F and Sb–F vibrations of the [PF_6_]^−^ and [SbF_6_]^−^ anions were assigned to 744 cm^−1^ and 645 cm^−1^, respectively, while the As–F vibration of the [AsF_6_]^−^ anion was tentatively assigned to 712 cm^−1^. All data agree well with the P–F, As–F, and Sb–F vibrations for [PF_6_]^−^ (756 cm^−1^), [AsF_6_]^−^ (689 cm^−1^), and [SbF_6_]^−^ anions (668 cm^−1^) published in the literature [33]. Assigning the As–F vibration in [^Me^CAACH][AsF_6_] was difficult because the more intense peak at 681 cm^−1^, which is in better agreement with the literature data, is likely a signal from the [^Me^CAACH]^+^ cation since it is present in the Raman spectra of all other aldiminium-based salts.

## 3. Materials and Methods

### 3.1. Reagents

Commercially available reagents BF_3_ (Union Carbide Austria GmbH, 99.5%), GeF_4_ (CERAC, Inc., Milwaukee, WI, USA, 99.99%), SbF_3_ (Alfa Aesar, Ward Hill, MA, USA, 99%) and elemental fluorine F_2_ (Solvay Fluor and Derivate GmbH, Neder-Over-Heembeek, Brussels, Belgium 99.98%) were used as received. Anhydrous HF (aHF, Linde, Dublin, Ireland, 99.995%) was dried by mixing with K_2_NiF_6_ (Advance Research Chemicals, Inc., Catoosa, OK, USA) before use. SiF_4_ was synthesized according to a modified procedure described in the literature [34]. SnF_4_ was synthesized by fluorination of SnF_2_ (Aldrich, Burlington, MA, USA, 99%) with F_2_ at room temperature in aHF. AsF_5_ and PF_5_ were prepared by pressure fluorination of As_2_O_3_ (Alfa Aesar, Ward Hill, MA, USA, 99%) and P_2_O_5_ (Sigma-Aldrich, Burlington, MA, USA, ≥98.0%) in a closed system as described previously [35]. [^Me^CAACH][(HCl)_0.5_Cl] and [^Me^CAACH][F(HF)_2_] were synthesized according to the procedures described in the literature [10,11]. MeCN (Honeywell, Charlotte, NC, USA, ≥99.9%) was purified using the Vigor solvent purification system. MeCN-d^3^ (Deutero, Kastellaun, Germany, 99.0%) was stored in the glovebox (M. Braun, Garching bei Munchen, Germany) over 3 Å molecular sieves before use. 

### 3.2. Caution

F_2_ and aHF are extremely corrosive and highly dangerous gases. They should be handled with care by an experienced experimenter in a well-ventilated hood. F_2_ is also a strong oxidizer that should be handled in nickel or copper equipment. All equipment used for reactions with F_2_ should be thoroughly cleaned, degreased, and fluorinated before use. The gases BF_3_, SiF_4_, GeF_4_, PF_5_, and AsF_5_ are also toxic. They readily hydrolyze in the presence of water to form HF. Take care to avoid inhalation and contact with skin. Always wear protective clothing, gloves, and a face mask when handling corrosive gases.

### 3.3. General

All syntheses were carried out under anhydrous conditions. Nonvolatile compounds were stored and handled in a glovebox (M. Braun) maintained below 0.1 ppm O_2_ and H_2_O, while gaseous and volatile compounds were handled via a nickel and polytetrafluoroethylene (PTFE) vacuum line. All reactions were performed in tetrafluoroethylene-hexafluoropropylene (FEP) reaction vessels equipped with PTFE valves. Before use, the vessels were passivated with F_2_ at 1 bar for 2 h and then evacuated. Crystallization of all newly prepared compounds proceeded by vapor diffusion. Approximately 20 mg of each salt was dissolved in 0.5 mL of solvent (usually DCM) in a small vial. The small vial was placed in a larger wide-neck vial containing 2.5 mL of antisolvent (usually cyclopentane) and capped with a screw cap. After a few days, small crystals formed in the small vial.

### 3.4. Syntheses

#### 3.4.1. [^Me^CAACH][SiF_5_]

[^Me^CAACH][F(HF)_2_] (200 mg, 0.579 mmol) was added to an FEP reaction vessel and dissolved in 5 mL of anhydrous MeCN. SiF_4_ gas (76 mg, 0.730 mmol) was condensed at −196 °C in the FEP reaction vessel. The reaction mixture was warmed to room temperature and stirred overnight. Then all volatiles were removed under reduced pressure of 10^−2^−10^−3^ bar to give a white solid. Crystallization by vapor diffusion using DCM as solvent and cyclopentane as antisolvent formed single crystals of [^Me^CAACH][SiF_5_] suitable for X-ray analysis. Yield: 192 mg (81%). ^1^H NMR (CD_3_CN, 25 °C, 600.06 MHz): δ 8.83 (s, 1H, C2−H), 7.62 (t, 1H, *J* = 7.8 Hz, p-ArH), 7.47 (d, 2H, *J* = 7.8 Hz, m-ArH), 2.72 (sept, 2H, *J* = 6.7 Hz, i-Pr-CH_3_), 2.46 (s, 2H, CH_2_), 1.60 (s, 6H, C5−CH_3_), 1.53 (s, 6H, C3−CH_3_), 1.35 (d, 6H, *J* = 6.7 Hz, i-Pr-CH_3_), 1.10 (d, 6H, *J* = 6.8 Hz, i-Pr-CH_3_). ^13^C{^1^H} NMR (CD_3_CN, 25 °C, 150.89 MHz): 192.1 (C2−H), 145.6 (ipso-ArC), 133.1 (p-ArC), 130.1 (o-ArC), 126.6 (m-ArC), 85.8 (C5), 48.9 (C3), 48.8 (CH_2_), 30.4 (i-Pr-CH), 28.5 (C3-CH_3_), 26.3 (i-Pr-CH_3_), 26.2 (C5-CH_3_), 22.2 (i-Pr-CH_3_). ^19^F NMR (CD_3_CN, 25 °C, 564.62 MHz): −137.53 (br, SiF_5_^−^). ^29^Si NMR (CD_3_CN, 25 °C, 119.22 MHz): −149.86 (SiF_5_^−^). Raman [ν(Si–F) range]: ν = 712 cm^−1^. Crystal Data for C_20_H_32_N∙SiF_5_ (M = 409.55 g/mol): monoclinic, space group P2_1_/n (no. 14), a = 10.7357(4) Å, b = 19.1191(7) Å, c = 10.8661(3) Å, β = 95.235(3)°, V = 2221.1(1) Å^3^, Z = 4, T = 150 K, μ(CuKα) = 1.342 mm^−1^, Dcalc = 1.225 g/cm^3^, 35,176 reflections measured (4.1° ≤ 2Θ ≤ 72.3°), 4372 unique (Rint = 0.0362, Rsigma = 0.0165) which were used in all calculations. The final R1 was 0.0486 (I > 2σ(I)) and wR2 was 0.1416 (all data).

#### 3.4.2. [^Me^CAACH][GeF_5_]

[^Me^CAACH][F(HF)_2_] (200 mg, 0.579 mmol) was added to an FEP reaction vessel and dissolved in 5 mL of anhydrous MeCN. GeF_4_ gas (92 mg, 0.619 mmol) was condensed at −196 °C in the FEP reaction vessel. The reaction mixture was warmed to room temperature and stirred overnight. Then, all volatiles were removed under reduced pressure of 10^−2^−10^−3^ bar to give a white solid. Crystallization by vapor diffusion using DCM as solvent and cyclopentane as antisolvent formed single crystals of [^Me^CAACH][GeF_5_] suitable for X-ray analysis. Yield: 253 mg (96%). ^1^H NMR (CD_3_CN, 25 °C, 600.06 MHz): δ 8.79 (s, 1H, C2−H), 7.61 (t, 1H, *J* = 7.8 Hz, p-ArH), 7.47 (d, 2H, *J* = 7.8 Hz, m-ArH), 2.72 (sept, 2H, *J* = 6.7 Hz, i-Pr-CH_3_), 2.45 (s, 2H, CH_2_), 1.60 (s, 6H, C5−CH_3_), 1.53 (s, 6H, C3−CH_3_), 1.35 (d, 6H, *J* = 6.7 Hz, i-Pr-CH_3_), 1.10 (d, 6H, *J* = 6.7 Hz, i-Pr-CH_3_). ^13^C{^1^H} NMR (CD_3_CN, 25 °C, 150.89 MHz): 192.1 (C2−H), 145.6 (ipso-ArC), 133.1 (p-ArC), 130.1 (o-ArC), 126.6 (m-ArC), 85.8 (C5), 48.9 (C3), 48.8 (CH_2_), 30.4 (i-Pr-CH), 28.5 (C3-CH_3_), 26.3 (i-Pr-CH_3_), 26.2 (C5-CH_3_), 22.2 (i-Pr-CH_3_). ^19^F NMR (CD_3_CN, 25 °C, 564.62 MHz): −137.45 (br, GiF_5_^−^). Raman [ν(Ge–F) range]: ν = 665 cm^−1^. Crystal Data for C_20_H_32_N∙GeF_5_ (M = 454.05 g/mol): monoclinic, space group P2_1_/n (no. 14), a = 10.8904(1) Å, b = 19.2640(2) Å, c = 10.9361(2) Å, β = 95.391(1)°, V = 2284.18(5) Å3, Z = 4, T = 150 K, μ(CuKα) = 2.219 mm^−1^, Dcalc = 1.320 g/cm^3^, 62,624 reflections measured (4.0° ≤ 2Θ ≤ 72.3°), 4493 unique (Rint = 0.0365, Rsigma = 0.0118) which were used in all calculations. The final R1 was 0.0425 (I > 2σ(I)) and wR2 was 0.1243 (all data).

#### 3.4.3. [^Me^CAACH][(THF)SnF_5_]

SnF_4_ (113 mg, 0.580 mmol) and [^Me^CAACH][F(HF)_2_] (200 mg, 0.579 mmol) were added to an FEP reaction vessel. Approximately 5 mL of aHF was condensed at −196 °C in the FEP vessel. The reaction mixture was warmed to room temperature and stirred overnight. Then, all volatiles were removed under reduced pressure of 10^−2^−10^−3^ bar to give a brown solid. In the glovebox, 5 mL of THF was added and a white precipitate was formed. The precipitate was separated from the solution, dried, and characterized as [^Me^CAACH][(THF)SnF_5_]. Yield: 298 mg (90%). ^1^H NMR (CD_3_CN, 25 °C, 600.06 MHz): δ 8.92 (s, 1H, C2−H), 7.61 (t, 1H, *J* = 7.8 Hz, p-ArH), 7.47 (d, 2H, *J* = 7.8 Hz, m-ArH), 3.64 (br, 4H, THF), 2.72 (sept, 2H, *J* = 6.7 Hz, i-Pr-CH_3_), 2.45 (s, 2H, CH_2_), 1.80 (s, 4H, THF), 1.61 (s, 6H, C5−CH_3_), 1.52 (s, 6H, C3−CH_3_), 1.35 (d, 6H, *J* = 6.7 Hz, i-Pr-CH_3_), 1.11 (d, 6H, *J* = 6.8 Hz, i-Pr-CH_3_). ^13^C{^1^H} NMR (CD_3_CN, 25 °C, 150.89 MHz): 192.5 (C2−H), 145.7 (ipso-ArC), 133.0 (p-ArC), 130.1 (o-ArC), 126.5 (m-ArC), 85.7 (C5), 68.5 (THF), 48.9 (C3), 48.9 (CH_2_), 30.4 (i-Pr-CH), 28.5 (C3-CH_3_), 26.3 (i-Pr-CH_3_), 26.2 (C5-CH_3_), 22.3 (i-Pr-CH_3_). ^19^F NMR (CD_3_CN, −23 °C, 564.62 MHz): −160.37 (d, 4F_cis_, _2_*J*_FF_ = 45.1 Hz, _1_*J*_119SnF_ = 1693 Hz), −170.20 (quint, 1F_trans_, _2_*J*_FF_ = 45.0 Hz, _1_*J*_119SnF_ = 1890 Hz). ^119^Sn NMR (CD_3_CN, −23 °C, 223.77 MHz): −789.93 (m, _1_*J*_119SnF_ = 1880 Hz, SnF_5_^−^). Raman [ν(Sn–F) range]: ν = 581 cm^−1^.

#### 3.4.4. [^Me^CAACH][PF_6_]

[^Me^CAACH][H_0.5_Cl_1.5_] (300 mg, 0.932 mmol) was added to an FEP reaction vessel. Approximately 5 mL of aHF and about 3 mmol of PF_5_ gas were condensed at −196 °C in the FEP reaction vessel. The reaction mixture was warmed to room temperature and stirred overnight. Then, all volatiles were removed under reduced pressure of 10^−2^−10^−3^ bar to give a white solid. Crystallization by vapor diffusion using DCM as solvent and cyclopentane as antisolvent formed single crystals of [^Me^CAACH][PF_6_] suitable for X-ray analysis. Yield: 346 mg (86%). ^1^H NMR (CD_3_CN, 25 °C, 600.06 MHz): δ 8.72 (s, 1H, C2−H), 7.61 (t, 1H, *J* = 7.8 Hz, p-ArH), 7.47 (d, 2H, *J* = 7.8 Hz, m-ArH), 2.72 (sept, 2H, *J* = 6.6 Hz, i-Pr-CH_3_), 2.46 (s, 2H, CH_2_), 1.60 (s, 6H, C5−CH_3_), 1.53 (s, 6H, C3−CH_3_), 1.35 (d, 6H, *J* = 6.5 Hz, i-Pr-CH_3_), 1.10 (d, 6H, *J* = 6.6 Hz, i-Pr-CH_3_). ^13^C{^1^H} NMR (CD_3_CN, 25 °C, 150.89 MHz): 191.8 (C2−H), 145.6 (ipso-ArC), 133.1 (p-ArC), 130.0 (o-ArC), 126.6 (m-ArC), 85.9 (C5), 48.8 (C3), 48.8 (CH_2_), 30.4 (i-Pr-CH), 28.5 (C3-CH_3_), 26.3 (i-Pr-CH_3_), 26.2 (C5-CH_3_), 22.2 (i-Pr-CH_3_). ^19^F NMR (CD_3_CN, 25 °C, 564.62 MHz): −72.84 (d, *J* = 706.7 Hz, PF_6_^−^). ^31^P NMR (CD_3_CN, 25 °C, 119.22 MHz): −146.09 (sept, *J* = 707.1 Hz, PF_6_^−^). Raman [ν(P–F) range]: ν = 744 cm^−1^.

#### 3.4.5. [^Me^CAACH][AsF_6_]

[^Me^CAACH][H_0.5_Cl_1.5_] (200 mg, 0.588 mmol) was added to an FEP reaction vessel. Approximately 5 mL of aHF and AsF_5_ gas (109 mg, 0.642 mmol) were condensed at −196 °C in the FEP reaction vessel. The reaction mixture was warmed to room temperature and stirred overnight. Then, all volatiles were removed under reduced pressure of 10^−2^−10^−3^ bar to give a white solid. Crystallization by vapor diffusion using DCM as solvent and cyclopentane as antisolvent formed single crystals of [^Me^CAACH][AsF_6_] suitable for X-ray analysis. Yield: 346 mg (86%). ^1^H NMR (CD_3_CN, 25 °C, 600.06 MHz): δ 8.72 (s, 1H, C2−H), 7.61 (t, 1H, *J* = 7.8 Hz, p-ArH), 7.48 (d, 2H, *J* = 7.9 Hz, m-ArH), 2.72 (sept, 2H, *J* = 6.7 Hz, i-Pr-CH_3_), 2.46 (s, 2H, CH_2_), 1.60 (s, 6H, C5−CH_3_), 1.53 (s, 6H, C3−CH_3_), 1.35 (d, 6H, *J* = 6.7 Hz, i-Pr-CH_3_), 1.10 (d, 6H, *J* = 6.8 Hz, i-Pr-CH_3_). ^13^C NMR (CD_3_CN, 25 °C, 150.89 MHz): 191.8 (C2−H), 145.6 (ipso-ArC), 133.1 (p-ArC), 130.0 (o-ArC), 126.6 (m-ArC), 85.9 (C5), 48.8 (C3), 48.8 (CH_2_), 30.4 (i-Pr-CH), 28.5 (C3-CH_3_), 26.3 (i-Pr-CH_3_), 26.2 (C5-CH_3_), 22.2 (i-Pr-CH_3_). ^19^F NMR (CD_3_CN, 25 °C, 564.62 MHz): −65.77 (m, *J* = 931.2 Hz, AsF_6_^−^). ^75^As NMR (CD_3_CN, 25 °C, 564.62 MHz): 5.45 (sept, *J* = 931.6 Hz, AsF_6_^−^). Raman [ν(As–F) range]: ν = 712 cm^−1^.

#### 3.4.6. [^Me^CAACH][SbF_6_]

SbF_3_ (155 mg, 0.867 mmol) was added to an FEP reaction vessel. Approximately 5 mL of aHF was condensed at −196 °C in the FEP reaction vessel. The mixture was allowed to react with F_2_ at room temperature until all the solid SbF_3_ was fluorinated to SbF_5_ and consequently dissolved. The solution was then quantitatively transferred to a second FEP reaction vessel loaded with [^Me^CAACH][F(HF)_2_] (300 mg, 0.868 mmol). The reaction mixture was stirred overnight. Thereafter, all volatiles were removed under reduced pressure of 10^−2^−10^−3^ bar to give a brown solid. Yield: 253 mg (96%). ^1^H NMR (CD_3_CN, 25 °C, 600.06 MHz): δ 8.71 (s, 1H, C2−H), 7.61 (t, 1H, *J* = 7.8 Hz, p-ArH), 7.48 (d, 2H, *J* = 7.8 Hz, m-ArH), 2.72 (sept, 2H, *J* = 6.7 Hz, i-Pr-CH_3_), 2.46 (s, 2H, CH_2_), 1.59 (s, 6H, C5−CH_3_), 1.53 (s, 6H, C3−CH_3_), 1.35 (d, 6H, *J* = 6.7 Hz, i-Pr-CH_3_), 1.10 (d, 6H, *J* = 6.8 Hz, i-Pr-CH_3_). ^13^C NMR (CD_3_CN, 25 °C, 150.89 MHz): 191.8 (C2−H), 145.6 (ipso-ArC), 133.1 (p-ArC), 130.1 (o-ArC), 126.6 (m-ArC), 86.0 (C5), 48.8 (C3), 48.8 (CH_2_), 30.4 (i-Pr-CH), 28.5 (C3-CH_3_), 26.3 (i-Pr-CH_3_), 26.2 (C5-CH_3_), 22.2 (i-Pr-CH_3_). ^19^F NMR (CD_3_CN, 25 °C, 564.62 MHz): −123.96 (m, *J* = 1936.3 Hz for ^121^Sb, and 1050.4 Hz for ^123^Sb, SbF_6_^−^). ^121^Sb NMR (CD_3_CN, 25 °C, 376.51 MHz): 86.29 (sept, *J* = 1934.68 Hz, SbF_6_^−^). Raman [ν(Sb–F) range]: ν = 645 cm^−1^.

#### 3.4.7. [^Me^CAACH][BF_4_]

[^Me^CAACH][H_0.5_Cl_1.5_] (308 mg, 0.906 mmol) was added to an FEP reaction vessel. Approximately 5 mL of aHF and about 3 mmol of BF_3_ gas were condensed at −196 °C in the FEP reaction vessel. The reaction mixture was warmed to room temperature and stirred overnight. Then, all volatiles were removed under reduced pressure of 10^−2^−10^−3^ bar to give a white solid. Crystallization by vapor diffusion using DCM as solvent and cyclopentane as antisolvent formed single crystals of [^Me^CAACH][BF_4_] suitable for X-ray analysis. Yield: 297 mg (88%). NMR data are in agreement with previously published data [9].

### 3.5. NMR Spectroscopy

Samples were prepared under inert atmosphere in a glovebox (M. Braun). NMR spectra were recorded in 5 mm glass NMR tubes with FEP inlays. Measurements were performed at the Slovenian NMR Centre (National Institute of Chemistry) using a Bruker AVANCE NEO 600 MHz NMR spectrometer (Bruker Corporation, Billerica, MA, USA). Chemical shifts of ^1^H and ^13^C were referenced to residual MeCN-d^3^ signals and reported relative to TMS (tetramethylsilane). The chemical shifts of ^19^F, ^29^Si, ^31^P, ^75^As, ^119^Sn, and ^121^Sb were calculated according to IUPAC guidelines and are given relative to CCl_3_F, Me_4_Si in CDCl_3_, H_3_PO_4_, NaAsF_6_ in MeCN-d^3^, Me_4_Sn in C_6_D_6_, and KSbCl_6_ in MeCN-d^3^ [36].

### 3.6. Crystal Structure Determination

Crystal data for all compounds were collected with a Gemini A diffractometer (Agilent Technologies, Santa Clara, CA, USA) equipped with an Atlas CCD detector using graphite-monochromated Cu Kα radiation at 150 K. The data were processed using the CrysAlisPro software package [37]. An analytical absorption correction was applied to all data sets [38]. Structures were solved using the SHELXT program [39]. Structure refinement was performed using the SHELXL software [40] implemented in the Olex2 program package [41]. The figures were created using Diamond [42].

CCDC 2286835-2286843 contains the supplementary crystallographic data for this work. These data can be obtained free of charge via http://www.ccdc.cam.ac.uk/conts/retrieving.html accessed on 24 August 2023 (or from the CCDC, 12 Union Road, Cambridge CB2 1EZ, UK; Fax: +44 1223 336033; E-mail: deposit@ccdc.cam.ac.uk).

### 3.7. Raman Spectroscopy

Samples were filled into 0.3 mm quartz capillaries under an inert atmosphere in a glovebox (M. Braun). Raman spectra were recorded using a Horiba Jobin Yvon Labram-HR spectrometer (HORIBA, Ltd., 2 Miyanohigashi, Kisshoin, Minami-ku Kyoto, 601-8510 Japan) coupled with an Olympus BXFM-ILHS microscope (Olympus Corporation, Shinjuku, Tokyo, Japan) at room temperature. Samples were excited with the 633 nm emission line of a He–Ne laser.

## 4. Conclusions

Aldiminium-based salts can be used as CAAC precursors or as unusual group transfer reagents. Since not many aldiminium-based compounds are yet known, we have carried out a systematic study on the synthesis of aldiminium-based salts of fluoroanions of groups 14 and 15. To achieve this goal, the reactivity of group 14 tetrafluorides (SiF_4_, GeF_4_, and SnF_4_) and group 15 pentafluorides (PF_5_, AsF_5_, and SbF_5_) was studied using the CAAC-based trifluoride reagent [^Me^CAACH][F(HF)_2_]. SiF_4_ and GeF_4_ react with [^Me^CAACH][F(HF)_2_] in MeCN to form [^Me^CAACH][SiF_5_] or [^Me^CAACH][GeF_5_]. The latter compound is only the second example of a discrete, structurally characterized [GeF_5_]^−^ anion. In a similar reaction, the SnF_4_ reacts with [^Me^CAACH][F(HF)_2_] in anhydrous HF most likely to give [^Me^CAACH][SnF_5_]. However, the interactions with all solvents used for crystallization led to the formation of octahedral species. This is also confirmed by the absence of crystal structures with a discrete [SnF_5_]^−^ anion. The compound was characterized in the form of [^Me^CAACH][(THF)SnF_5_] and [^Me^CAACH][(dioxane)SnF_5_] after dissolving [^Me^CAACH][SnF_5_] in THF and dioxane, respectively. Both salts contain discrete, octahedrally coordinated tin fluoride anions with solvent molecules and are the first examples of these types of anions. [^Me^CAACH][PF_6_] and [^Me^CAACH][AsF_6_] were synthesized in one pot by the reaction of PF_5_ or AsF_5_ gasses with [^Me^CAACH][F(HF)_2_] prepared in situ from [^Me^CAACH][Cl(HCl)_0.5_] and aHF. Finally, [^Me^CAACH][SbF_6_] was prepared by the reaction of freshly prepared SbF_5_ with [^Me^CAACH][F(HF)_2_] in aHF. All the newly prepared compounds with discrete [SiF_5_]^−^, [GeF_5_]^−^, [(THF)SnF_5_]^−^, [(dioxane)SnF_5_]^−^, [PF_6_]^−^, [AsF_6_]^−^ and [SbF_6_]^−^ anions were structurally and spectroscopically characterized by X-ray single-crystal structure analysis, NMR and Raman spectroscopy. In conclusion, the stabilization of the rare 5- and 6- coordinated main group fluoroanions would not be possible without the aldiminium-based cation [^Me^CAACH]^+^. It was found to stabilize the discrete anions and prevent oligomerization of the fluorides used.

## Figures and Tables

**Table 1:** Selected NMR peaks of fluoro-compounds in MeCN-d3.

Compound	*δ*(^19^F) M–F	Isotope (M)	*δ*(Isotope) M–F
[^Me^CAACH][BF_4_]	−151.94 (s)	^11^B	−1.23 (s)
[^Me^CAACH][SiF_5_]	−137.53 (br)	^29^Si	−149.86 (s)
[^Me^CAACH][GeF_5_]	−137.45 (br)	^74^Ge	na
[^Me^CAACH][(THF)SnF_5_]	−160.37 (d), −170.20 (quint)	^119^Sn	−789.93 (m)
[^Me^CAACH][PF_6_]	−72.84 (d)	^31^P	−146.09 (sept)
[^Me^CAACH][AsF_6_]	−65.77 (m)	^75^As	5.45 (sept)
[^Me^CAACH][SbF_6_]	−123.96 (m)	^121^Sb	86.29 (sept)

**Table 2:** Selected Raman peaks of fluoro-compounds.

Compound	Raman *ν* M–F (cm^−1^)	Isotope (M)
[^Me^CAACH][BF_4_]	na	^11^B
[^Me^CAACH][SiF_5_]	712	^29^Si
[^Me^CAACH][GeF_5_]	665	^74^Ge
[^Me^CAACH][(THF)SnF_5_]	581	^119^Sn
[^Me^CAACH][PF_6_]	744	^31^P
[^Me^CAACH][AsF_6_]	712	^75^As
[^Me^CAACH][SbF_6_]	645	^121^Sb

## Data Availability

The data presented in this study are available on request from the corresponding author.

## Supplementary Materials

The following supporting information can be downloaded at: https://www.mdpi.com/article/10.3390/molecules28176270/s1, Figure S1. ^1^H NMR spectrum of [^Me^CAACH][BF_4_] in acetonitrile solution. Figure S2. ^13^C{^1^H} NMR spectrum of [^Me^CAACH][BF_4_] in acetonitrile solution. Figure S3. ^19^F NMR spectrum of [^Me^CAACH][BF_4_] in acetonitrile solution. Figure S4. ^11^B NMR spectrum of [^Me^CAACH][BF_4_] in acetonitrile solution. Figure S5. ^1^H NMR spectrum of [^Me^CAACH][SiF_5_] in acetonitrile solution. Figure S6. ^13^C{^1^H} NMR spectrum of [^Me^CAACH][SiF_5_] in acetonitrile solution. Figure S7. ^19^F NMR spectrum of [^Me^CAACH][SiF_5_] in acetonitrile solution. Figure S8. ^29^Si NMR spectrum of [^Me^CAACH][SiF_5_] in acetonitrile solution. Figure S9. ^1^H NMR spectrum of [^Me^CAACH][GeF_5_] in acetonitrile solution. Figure S10. ^13^C{^1^H} NMR spectrum of [^Me^CAACH][GeF_5_] in acetonitrile solution. Figure S11. ^19^F NMR spectrum of [^Me^CAACH][GeF_5_] in acetonitrile solution. Figure S12. ^1^H NMR spectrum of [^Me^CAACH][(THF)SnF_5_] in acetonitrile solution. Figure S13. ^13^C{^1^H} NMR spectrum of [^Me^CAACH][(THF)SnF_5_] in acetonitrile solution. Figure S14. ^19^F NMR spectrum of [^Me^CAACH][(THF)SnF_5_] in acetonitrile solution. Figure S15. ^119^Sn NMR spectrum of [^Me^CAACH][(THF)SnF_5_] in acetonitrile solution. Figure S16. ^1^H NMR spectrum of [^Me^CAACH][PF_6_] in acetonitrile solution. Figure S17. ^13^C{^1^H} NMR spectrum of [^Me^CAACH][PF_6_] in acetonitrile solution. Figure S18. ^19^F NMR spectrum of [^Me^CAACH][PF_6_] in acetonitrile solution. Figure S19. ^31^P NMR spectrum of [^Me^CAACH][PF_6_] in acetonitrile solution. Figure S20. ^1^H NMR spectrum of [^Me^CAACH][AsF_6_] in acetonitrile solution. Figure S21. ^13^C{^1^H} NMR spectrum of [^Me^CAACH][AsF_6_] in acetonitrile solution. Figure S22. ^19^F NMR spectrum of [^Me^CAACH][AsF_6_] in acetonitrile solution. Figure S23. ^75^As NMR spectrum of [^Me^CAACH][AsF_6_] in acetonitrile solution. Figure S24. ^1^H NMR spectrum of [^Me^CAACH][SbF_6_] in acetonitrile solution. Figure S25. ^13^C{^1^H} NMR spectrum of [^Me^CAACH][SbF_6_] in acetonitrile solution. Figure S26. ^19^F NMR spectrum of [^Me^CAACH][SbF_6_] in acetonitrile solution. Figure S27. ^19^F NMR spectrum of [^Me^CAACH][SbF_6_] in acetonitrile solution. Figure S28. Raman spectra of [^Me^CAACH][SiF_5_], [^Me^CAACH][GeF_5_], and [^Me^CAACH][(THF)SnF_5_]. Figure S29. Raman spectra of [^Me^CAACH][PF_6_], [^Me^CAACH][AsF_6_], and [^Me^CAACH][SbF_6_]. Figure S30. Raman spectra of [^Me^CAACH][BF_4_]. Table S1. Selected crystal data for [^Me^CAACH][SiF_5_], [^Me^CAACH][GeF_5_], and [^Me^CAACH][(THF)SnF_5_]. Table S2. Selected crystal data for [^Me^CAACH][(dioxane)SnF_5_]∙dioxane, [^Me^CAACH][PF_6_], and [^Me^CAACH][AsF_6_]. Table S3. Selected crystal data for [^Me^CAACH][BF_4_], [^Me^CAACH][Cl], and [^Me^CAACH][OTf]. Table S4. Selected bond lengths (Å) and bond angles (°) for [^Me^CAACH][SiF_5_], [^Me^CAACH][GeF_5_], and [^Me^CAACH][(THF)SnF_5_]. Table S5. Selected bond lengths (Å) and bond angles (°) for [^Me^CAACH][(dioxane)SnF_5_]∙dioxane, [^Me^CAACH][PF_6_] and [^Me^CAACH][AsF_6_]. Table S6. Selected bond lengths (Å) and bond angles (°) for [^Me^CAACH][BF_4_], [^Me^CAACH][Cl], and [^Me^CAACH][OTf]. Figure S31. Structure of the disordered [^Me^CAACH][SiF_5_]. Figure S32. Structure of the disordered [^Me^CAACH][GeF_5_]. Figure S33. Crystal structure of [^Me^CAACH][(dioxane)SnF_5_]∙dioxane with solvent molecules. Figure S34. Structure of the disordered [^Me^CAACH][BF_4_]. Figure S35. Structure of the asymmetric unit of [^Me^CAACH][OTf].

## Sample Availability

Samples of the reported compounds are available from the authors upon reasonable request.
