# Supplementary material for: Aldiminium Cations as Countercations to Discrete Main Group Fluoroanions
Source: Molecules. 2023 Aug 27;28(17):6270. doi: 10.3390/molecules28176270 (PMC10489094; doi:10.3390/molecules28176270)
Supplement: Supplementary file 1 [file molecules-28-06270-s001.zip › molecules-2570017-supplementary.pdf]

Supporting information

Aldiminium Cations as Countercations to Discrete Main Group Fluoroanions

Evelin Gruden,[1] and Gašper Tavčar[1,*]

[1] Department of Inorganic Chemistry and Technology, Jožef Stefan Institute, Jamova 39, 1000 Ljubljana, Slovenia

* Correspondence: gasper.tavcar@ijs.si (G.T)

Table of content

S1 NMR spectroscopy 2

S1.1 [^{Me}CAACH][BF_4] 2

S1.2 [^{Me}CAACH][SiF_5] 4

S1.3 [^{Me}CAACH][GeF_5] 6

S1.4 [^{Me}CAACH][(THF)SnF_5] 8

S1.5 [^{Me}CAACH][PF_6] 10

S1.6 [^{Me}CAACH][AsF_6] 12

S1.7 [^{Me}CAACH][SbF_6] 14

S2 Raman spectroscopy 16

S3 Crystal Structure Data 18

S3.1 Crystal structures of selected compounds 24

S1 NMR spectroscopy

S1.1 [^{Me}CAACH][BF_4]

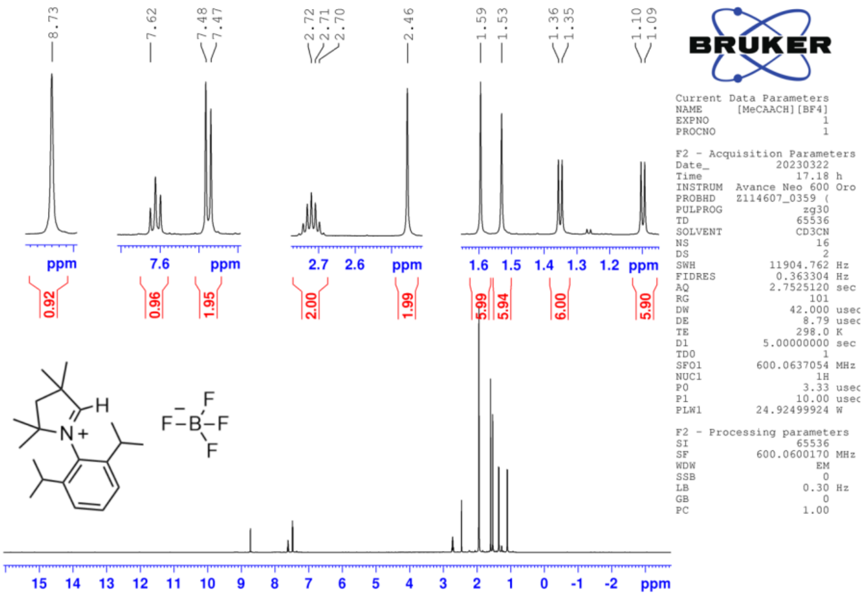

Figure S1. ^{1}H NMR spectrum of [^{Me}CAACH][BF_4] in acetonitrile solution.

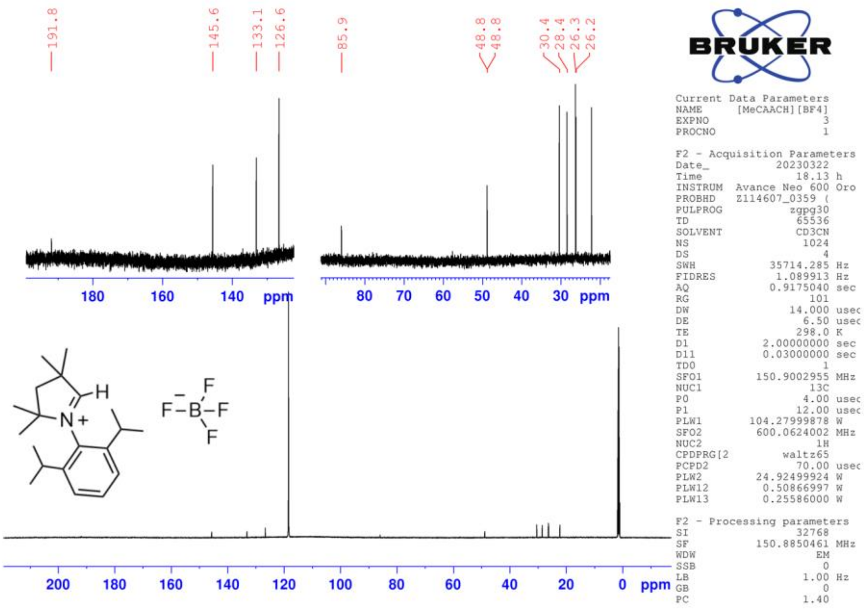

Figure S2. $^{13}C\{^{1}H\}$ NMR spectrum of [^{Me}CAACH][BF_4] in acetonitrile solution.

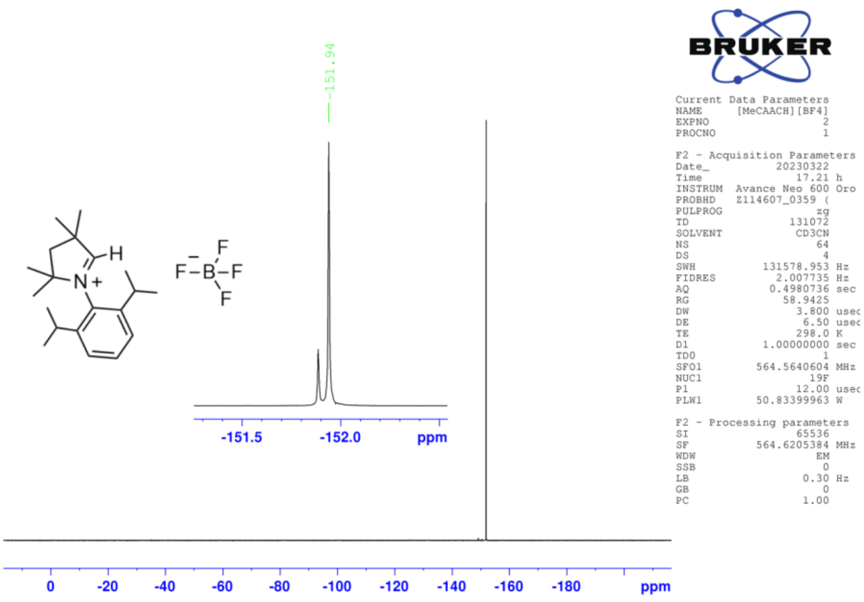

Figure S3. ^{19}F NMR spectrum of [^{Me}CAACH][BF_4] in acetonitrile solution.

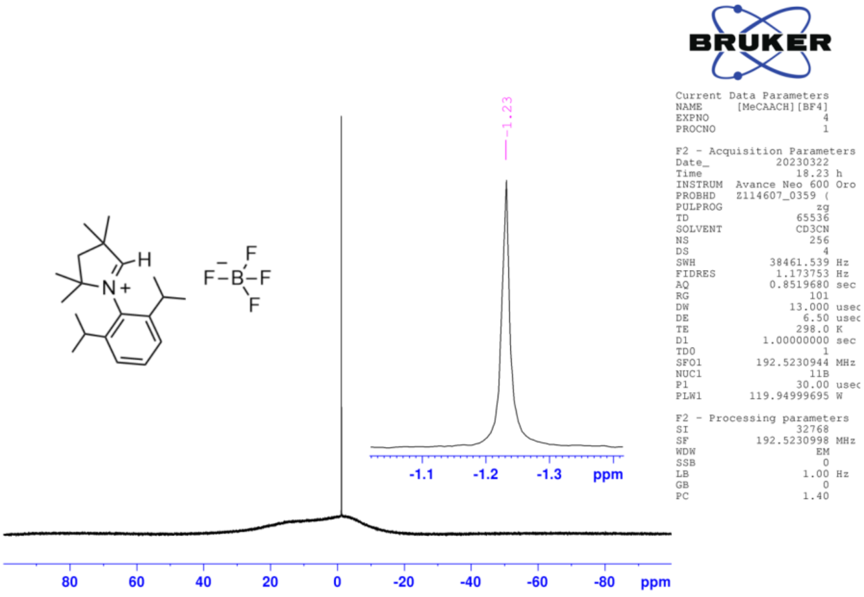

Figure S4. ^{11}B NMR spectrum of [^{Me}CAACH][BF_4] in acetonitrile solution.

S1.2 [^{Me}CAACH][SiF$_5$]

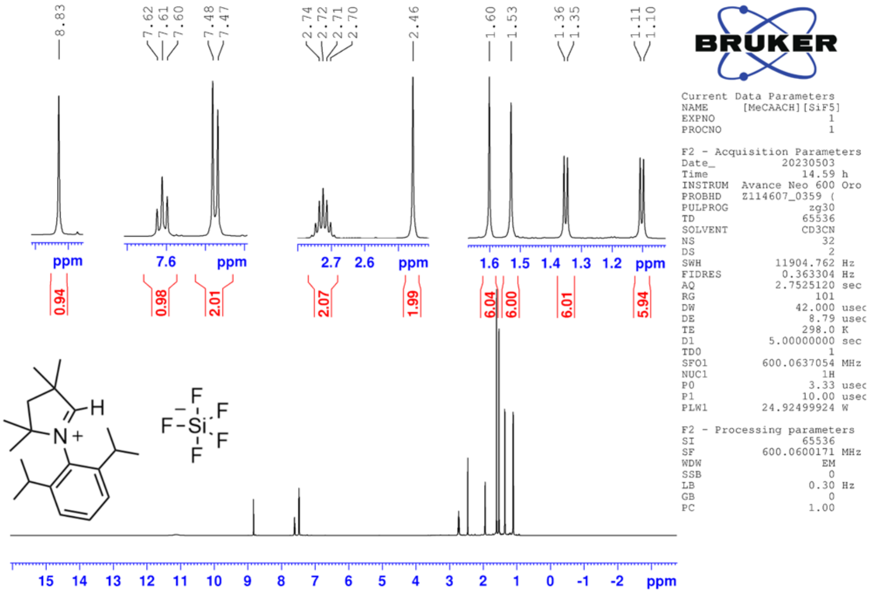

Figure S5. ^{1}H NMR spectrum of [^{Me}CAACH][SiF$_5$] in acetonitrile solution.

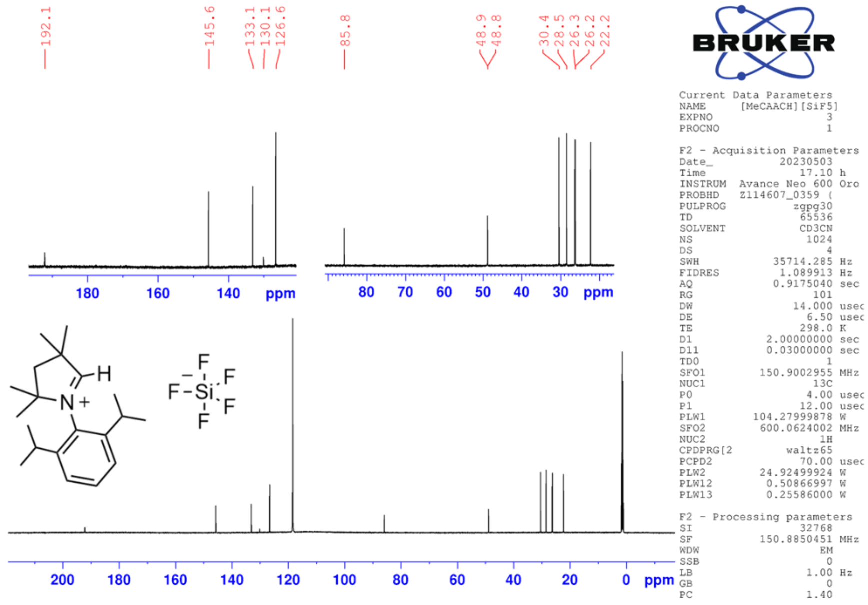

Figure S6. ^{13}C{^{1}H} NMR spectrum of [^{Me}CAACH][SiF$_5$] in acetonitrile solution.

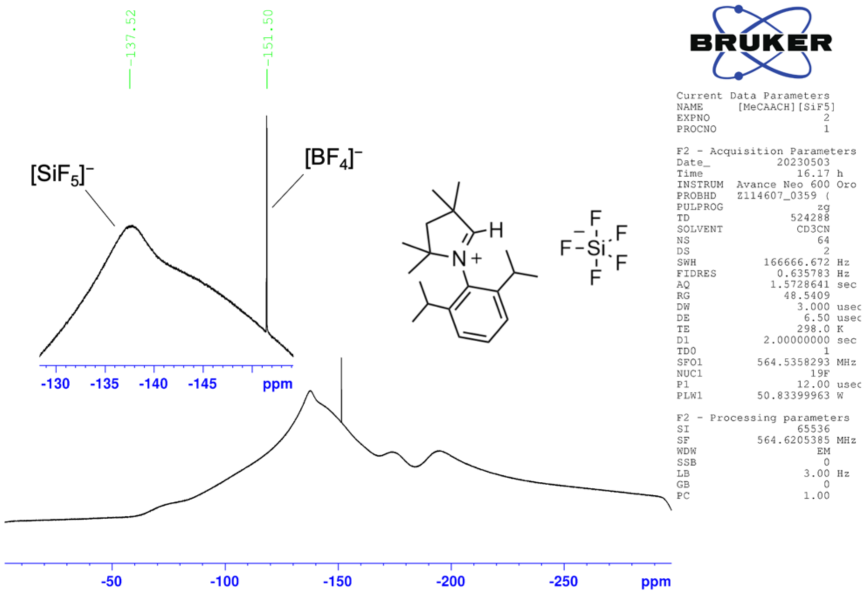

Figure S7. ^{19}F NMR spectrum of [^{Me}CAACH][SiF_5] in acetonitrile solution. The signal for [BF_4]$^-$ anions is visible due to the contamination of the reaction mixture with residual BF_3 gas in the vacuum line.

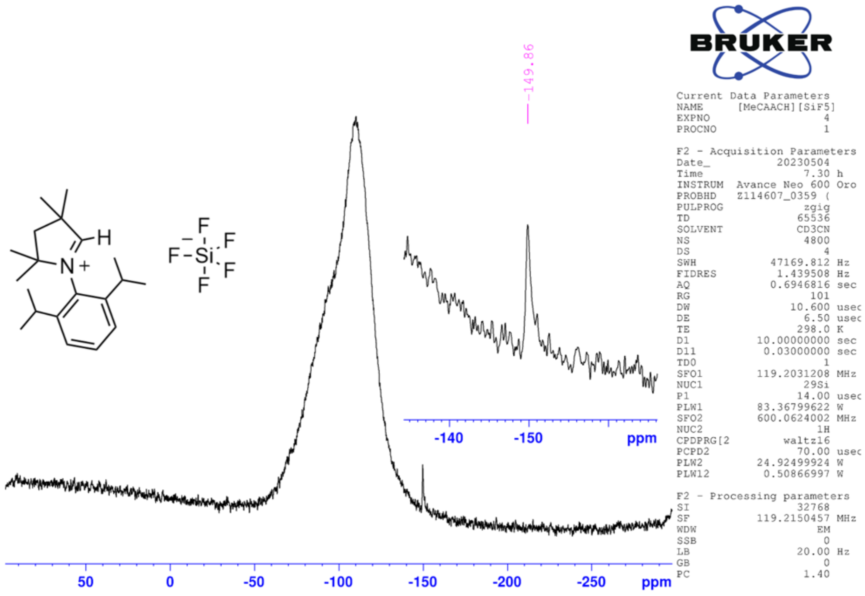

Figure S8. ^{29}Si NMR spectrum of [^{Me}CAACH][SiF_5] in acetonitrile solution.

S1.3 [^{Me}CAACH][GeF$_5$]

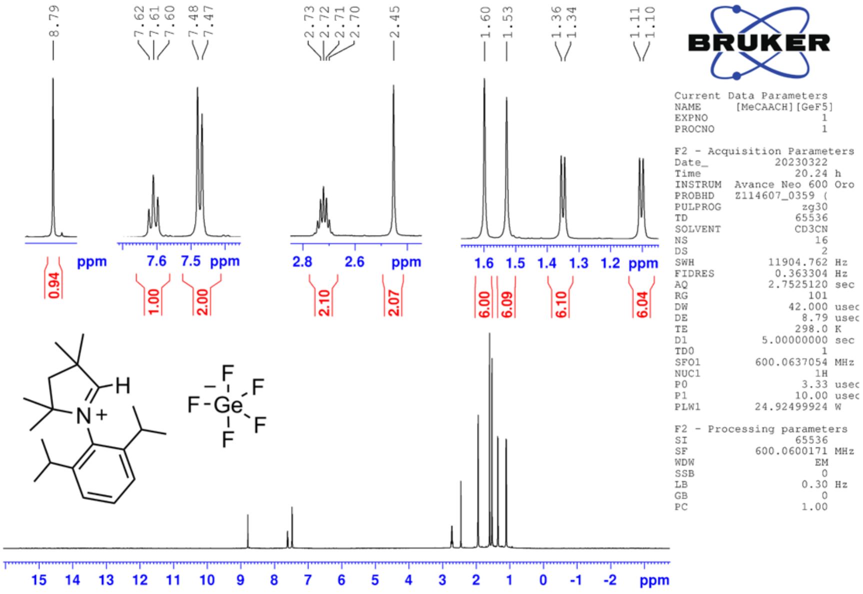

Figure S9. ^{1}H NMR spectrum of [^{Me}CAACH][GeF$_5$] in acetonitrile solution.

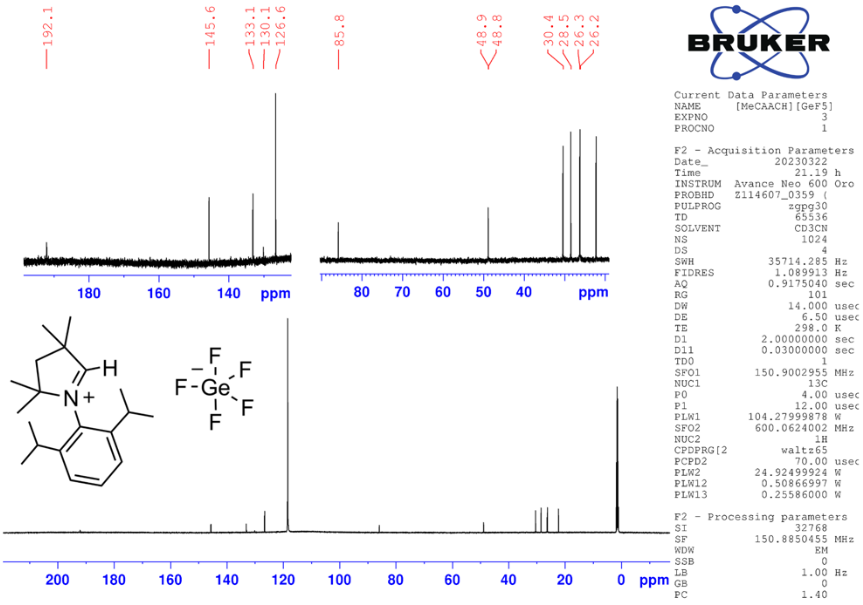

Figure S10. ^{13}C{^{1}H} NMR spectrum of [^{Me}CAACH][GeF$_5$] in acetonitrile solution.

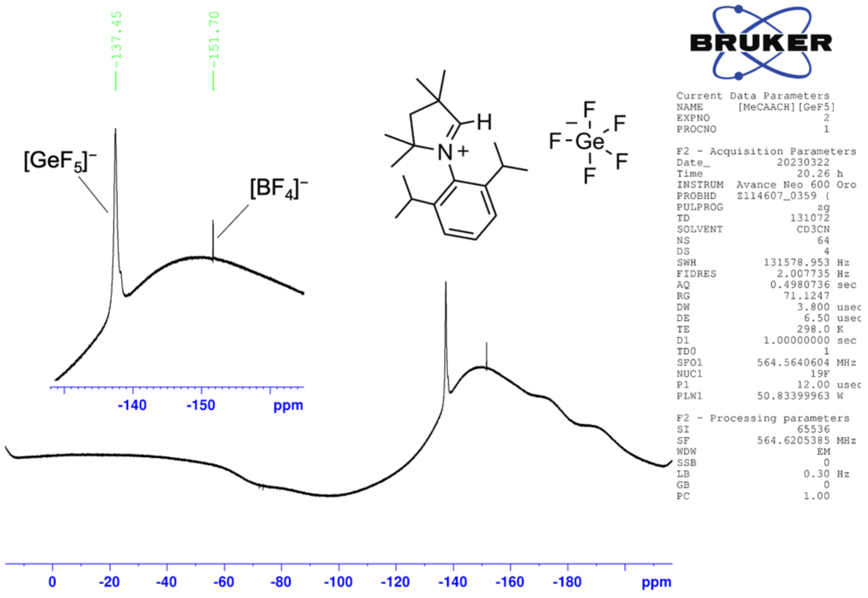

Figure S11. ^{19}F NMR spectrum of [^{Me}CAACH][GeF_5] in acetonitrile solution. The signal for [BF_4]$^-$ anions is visible due to the contamination of the reaction mixture with residual BF_3 gas in the vacuum line.

S1.4 [^{Me}CAACH][(THF)SnF$_5$]

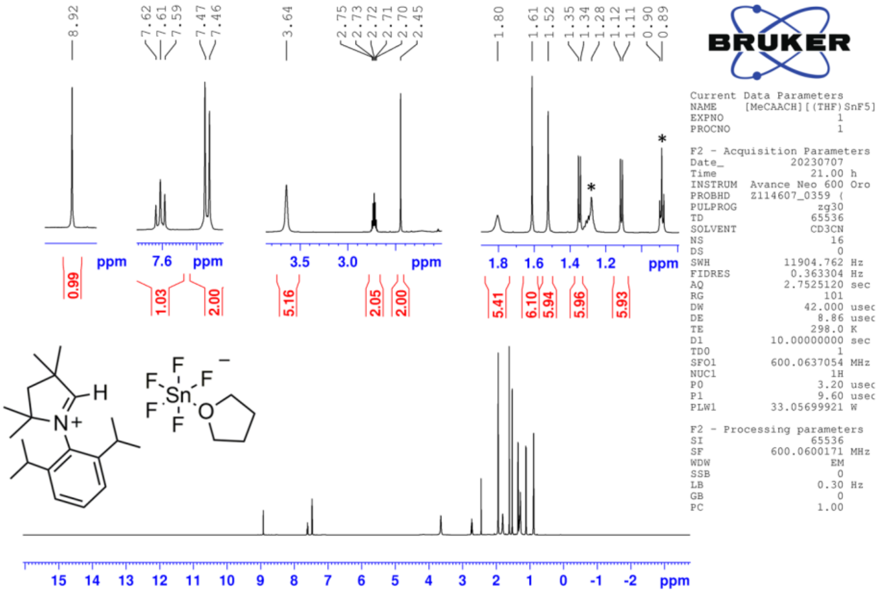

Figure S12. ^{1}H NMR spectrum of [^{Me}CAACH][(THF)SnF$_5$] in acetonitrile solution. The sample contains small amounts of residual THF and hexane. This is due to the fact, that the sample used was crystallized from the mixture of THF and hexane. Peaks at 1.28 ppm (m, 8H) and 0.89 ppm (t, 6H, J = 7.0 Hz) marked with * belong to hexane.

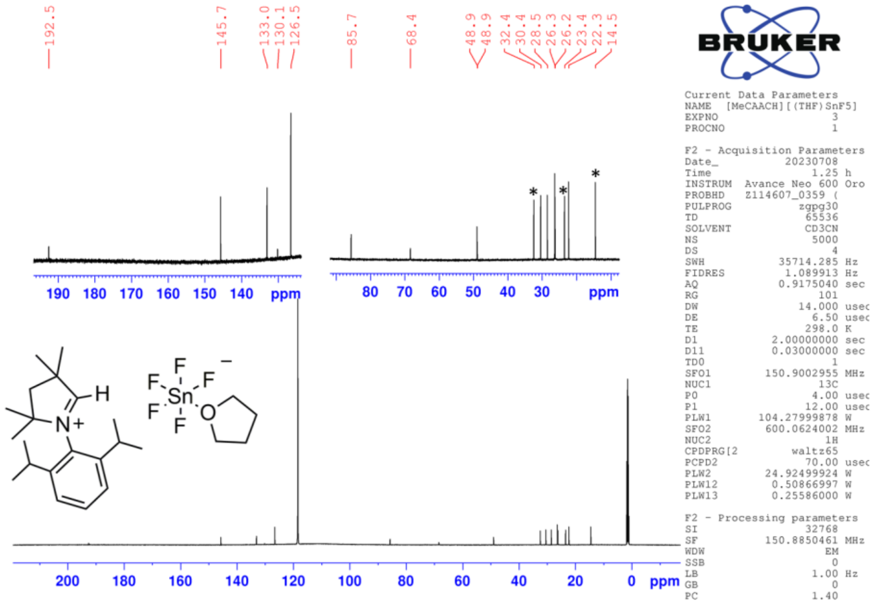

Figure S13. ^{13}C{^{1}H} NMR spectrum of [^{Me}CAACH][(THF)SnF$_5$] in acetonitrile solution. The sample contains small amounts of residual THF and hexane. This is due to the fact, that the sample used was crystallized from the mixture of THF and hexane. Peaks at 32.4 ppm, 23.4 ppm and 14.5 ppm marked with * belong to hexane.

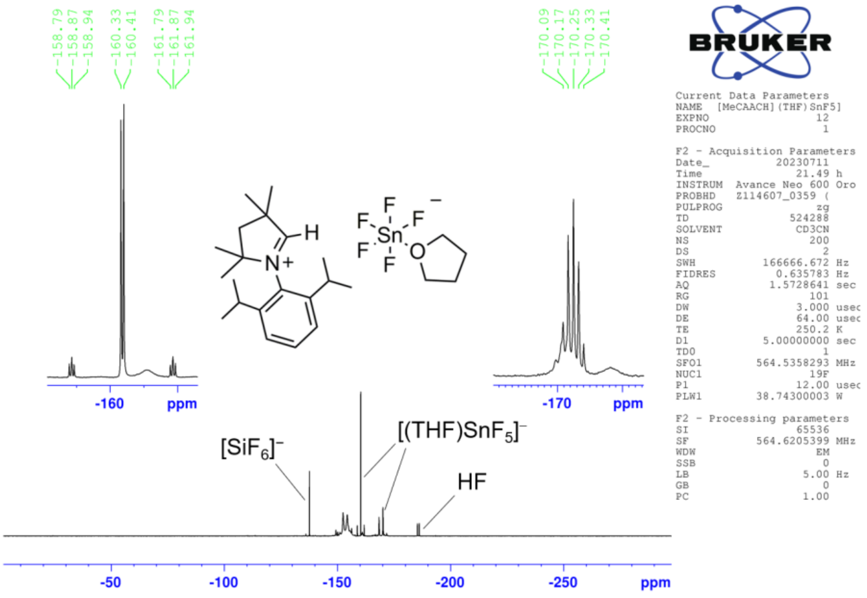

Figure S14. ^{19}F NMR spectrum of [^{Me}CAACH][(THF)SnF_5] in acetonitrile solution. The sample used for NMR measurements was crystallized from the mixture of THF and hexane in a glass vial. Because of the reaction of [SnF_5]$^-$ with glass, signals for HF (−185.84 ppm, d, J = 465.3 Hz), [SiF_6]$^{2-}$ anions (−137.74 ppm), and many other side products are visible. Unfortunately, we were not able to characterize the side products.

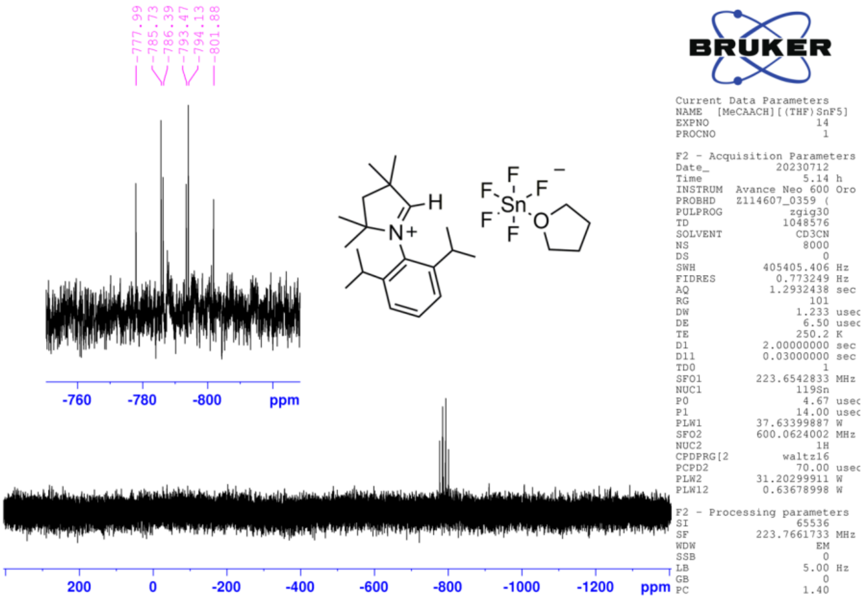

Figure S15. ^{119}Sn NMR spectrum of [^{Me}CAACH][(THF)SnF_5] in acetonitrile solution.

S1.5 [^{Me}CAACH][PF_6]

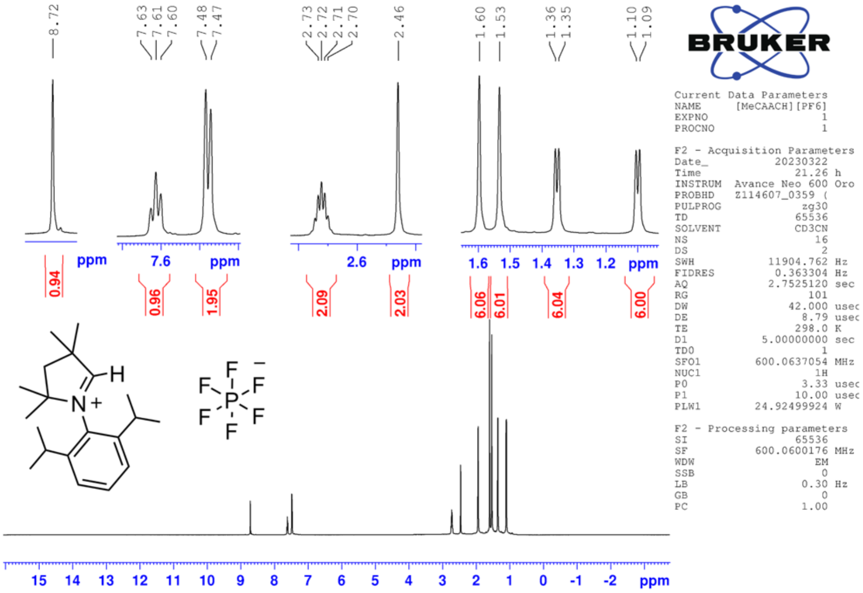

Figure S16. ^{1}H NMR spectrum of [^{Me}CAACH][PF_6] in acetonitrile solution.

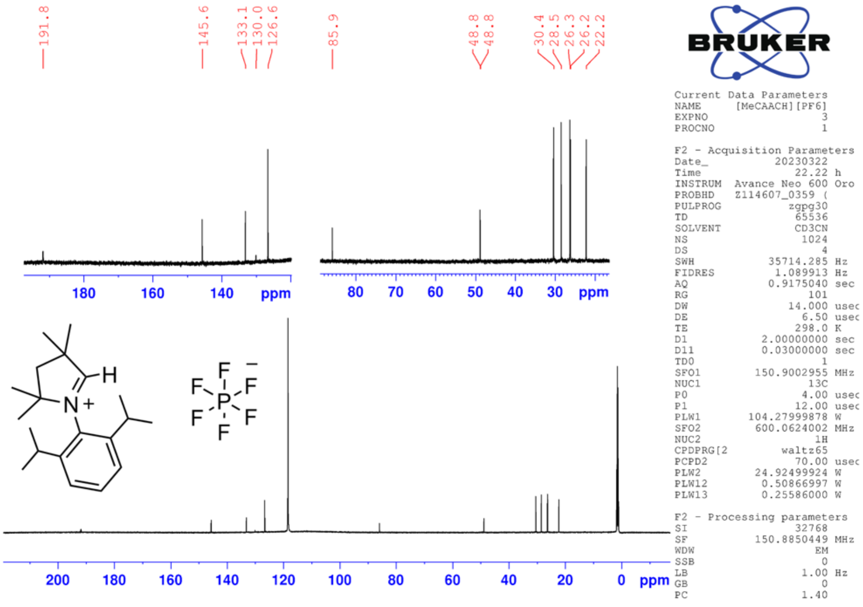

Figure S17. $^{13}C\{^{1}H\}$ NMR spectrum of [^{Me}CAACH][PF_6] in acetonitrile solution.

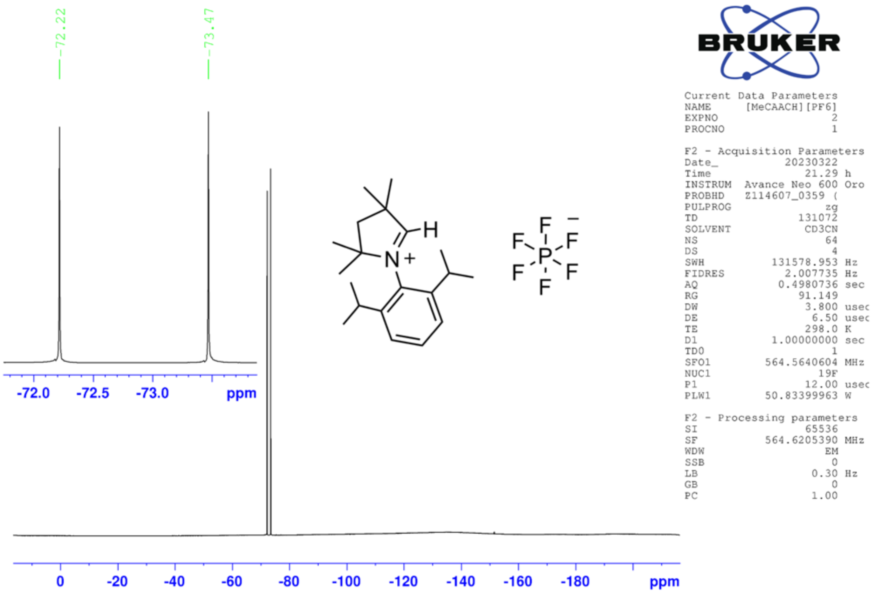

Figure S18. ^{19}F NMR spectrum of [^{Me}CAACH][PF_6] in acetonitrile solution.

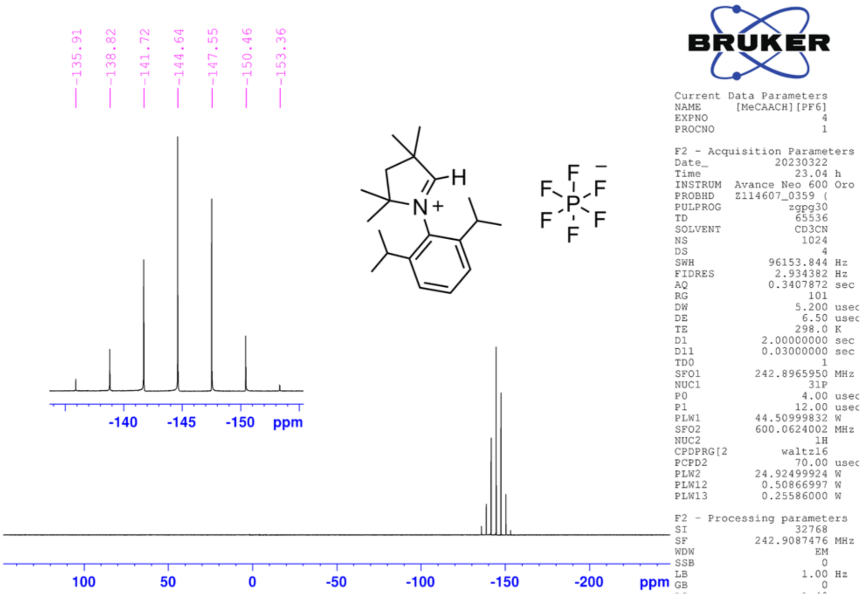

Figure S19. ^{31}P NMR spectrum of [^{Me}CAACH][PF_6] in acetonitrile solution.

S1.6 [^{Me}CAACH][AsF$_6$]

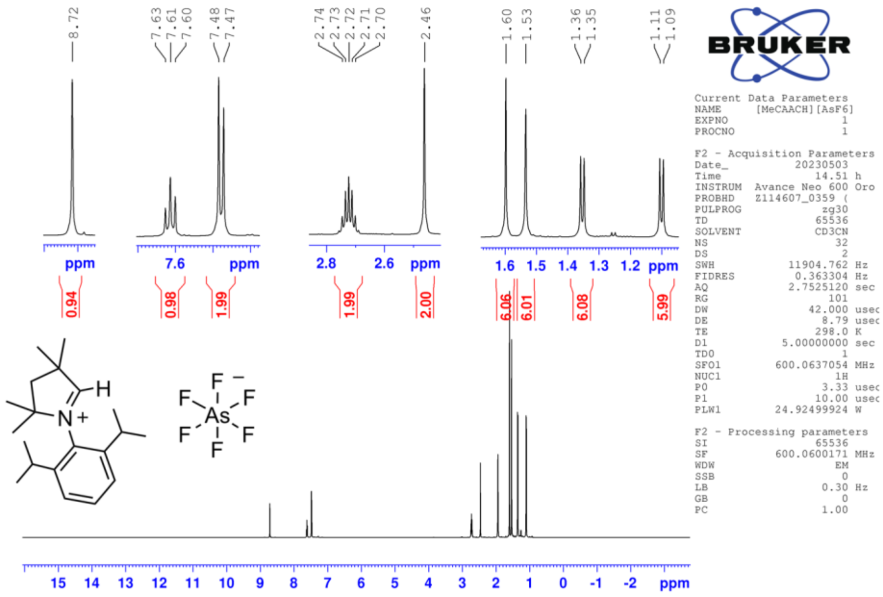

Figure S20. ^{1}H NMR spectrum of [^{Me}CAACH][AsF$_6$] in acetonitrile solution.

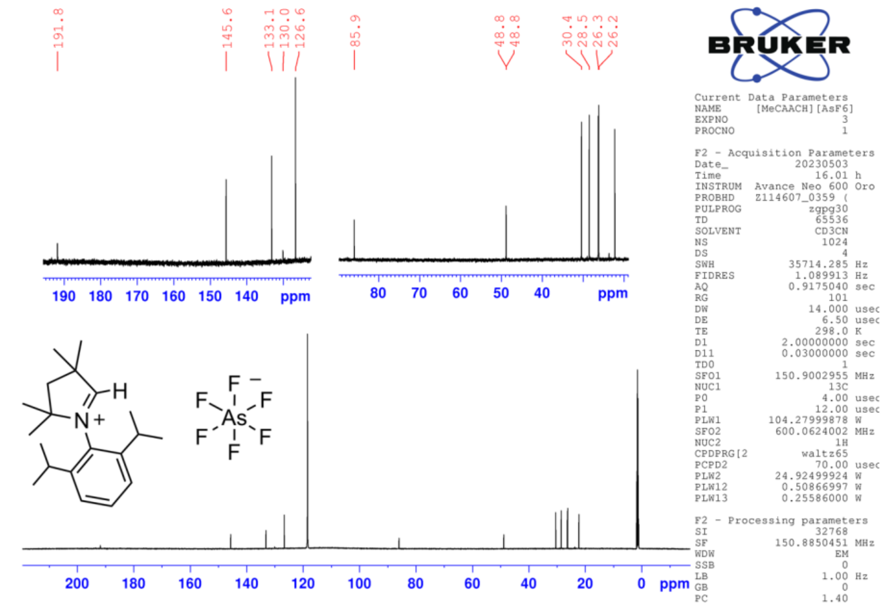

Figure S21. ^{13}C{^{1}H} NMR spectrum of [^{Me}CAACH][AsF$_6$] in acetonitrile solution.

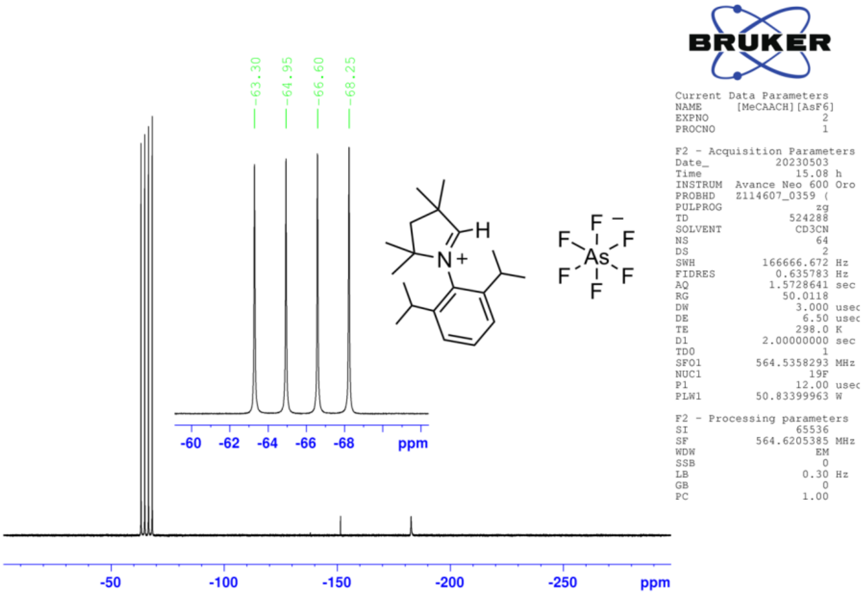

Figure S22. ^{19}F NMR spectrum of [^{Me}CAACH][AsF_6] in acetonitrile solution.

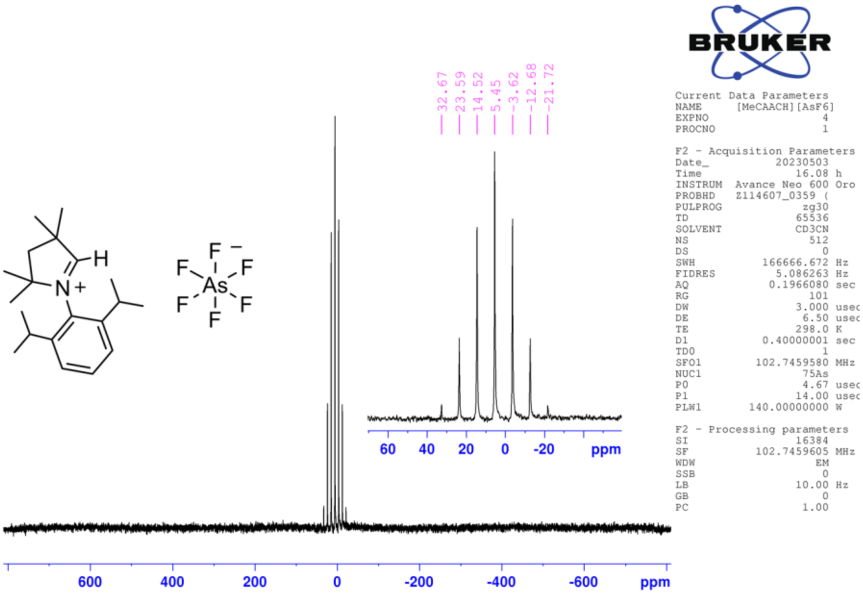

Figure S23. ^{75}As NMR spectrum of [^{Me}CAACH][AsF_6] in acetonitrile solution.

S1.7 [^{Me}CAACH][SbF$_6$]

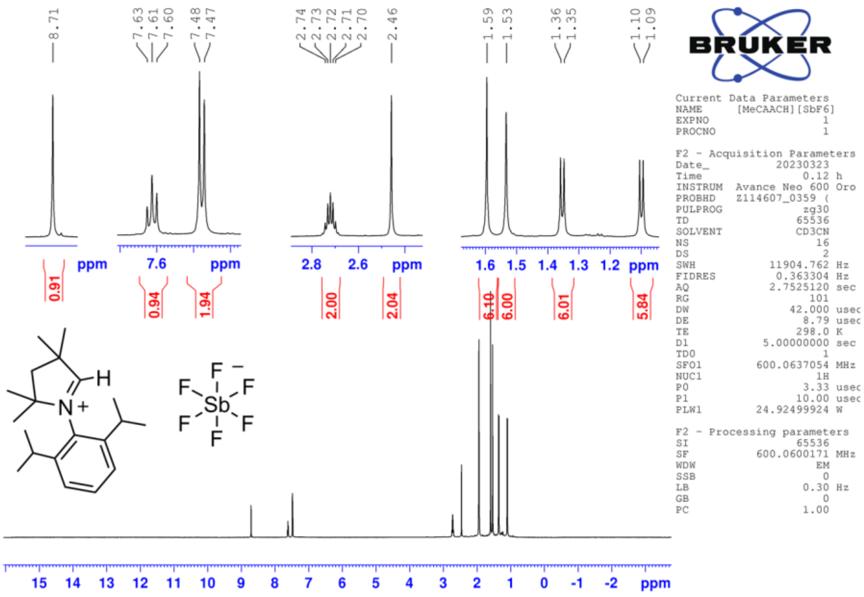

Figure S24. ^{1}H NMR spectrum of [^{Me}CAACH][SbF$_6$] in acetonitrile solution.

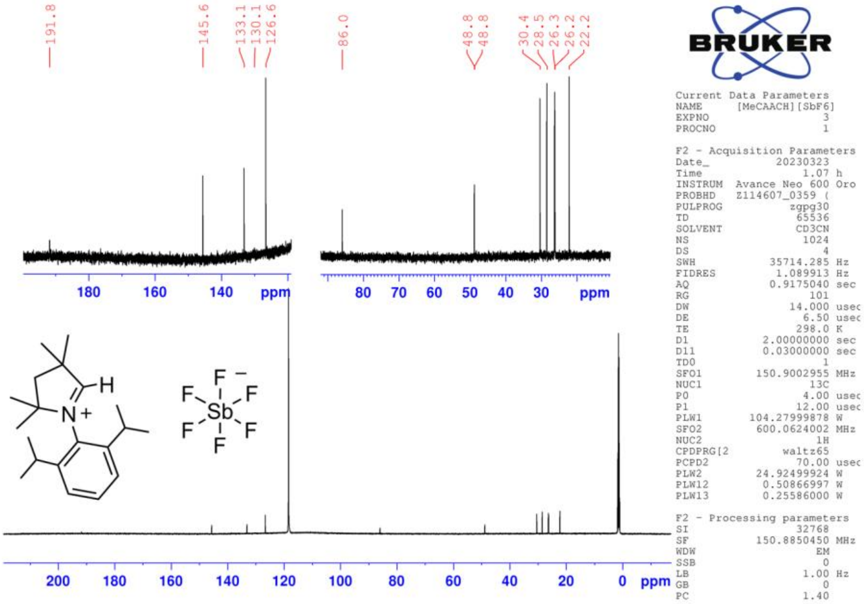

Figure S25. ^{13}C{^{1}H} NMR spectrum of [^{Me}CAACH][SbF$_6$] in acetonitrile solution.

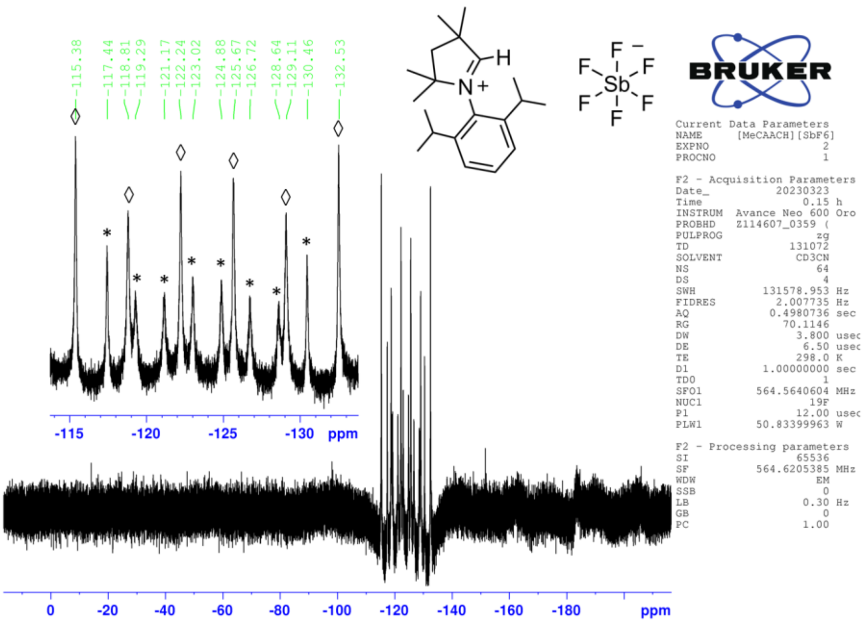

Figure S26. ^{19}F NMR spectrum of [^{Me}CAACH][SbF_6] in acetonitrile solution. Peaks marked with ◊ belong to anions with ^{121}Sb isotope, while the peaks marked with * belong to anions with ^{123}Sb isotope.

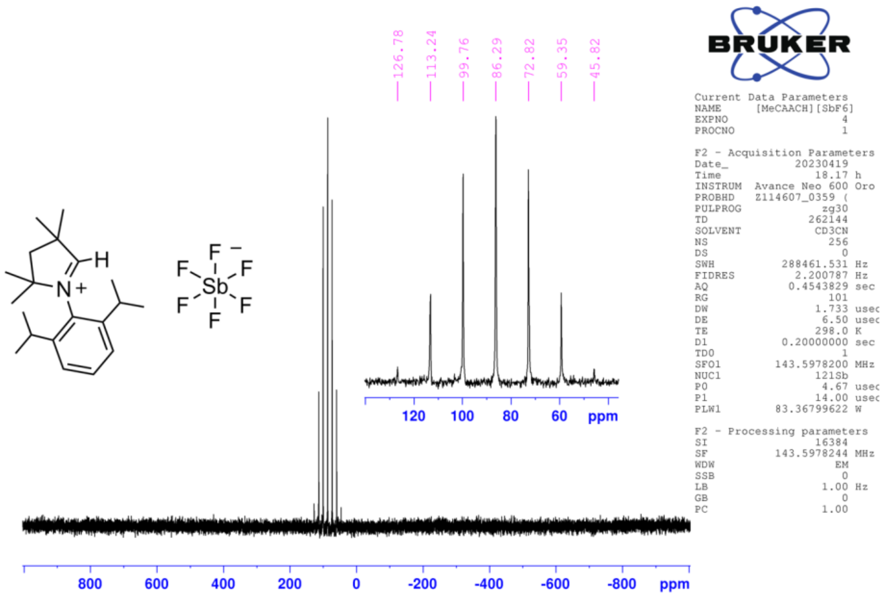

Figure S27. ^{19}F NMR spectrum of [^{Me}CAACH][SbF_6] in acetonitrile solution.

S2 Raman spectroscopy

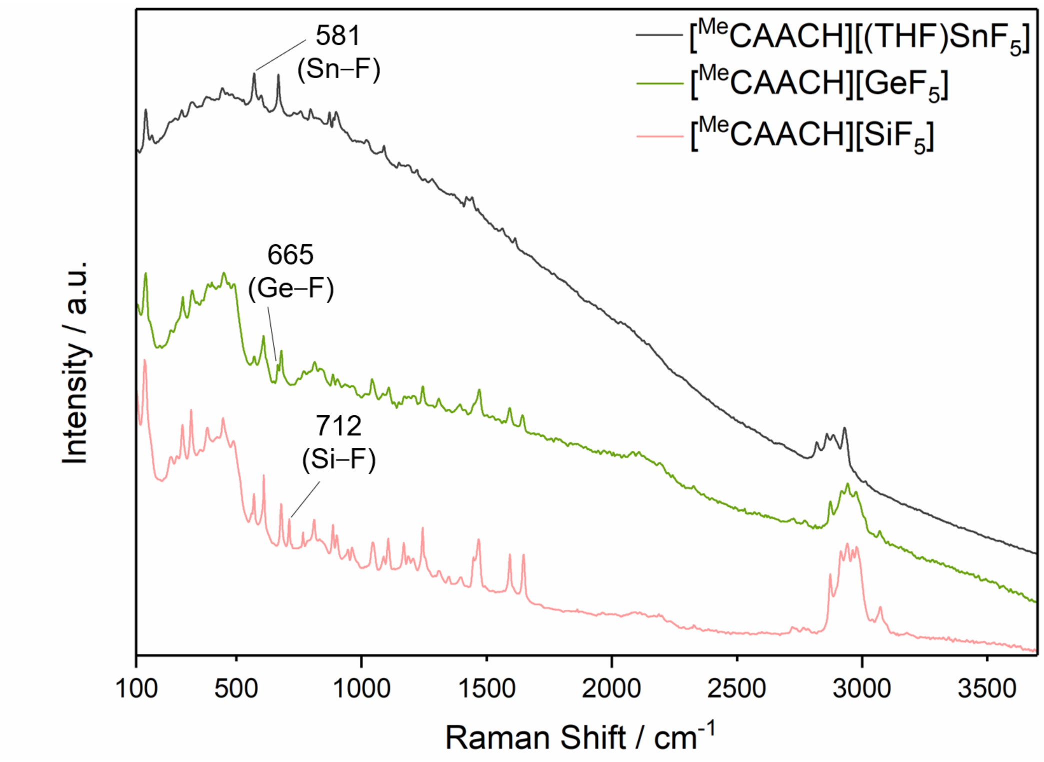

Figure S28. Raman spectra of [[Me]CAACH][SiF_5], [[Me]CAACH][GeF_5] and [[Me]CAACH][(THF)SnF_5].

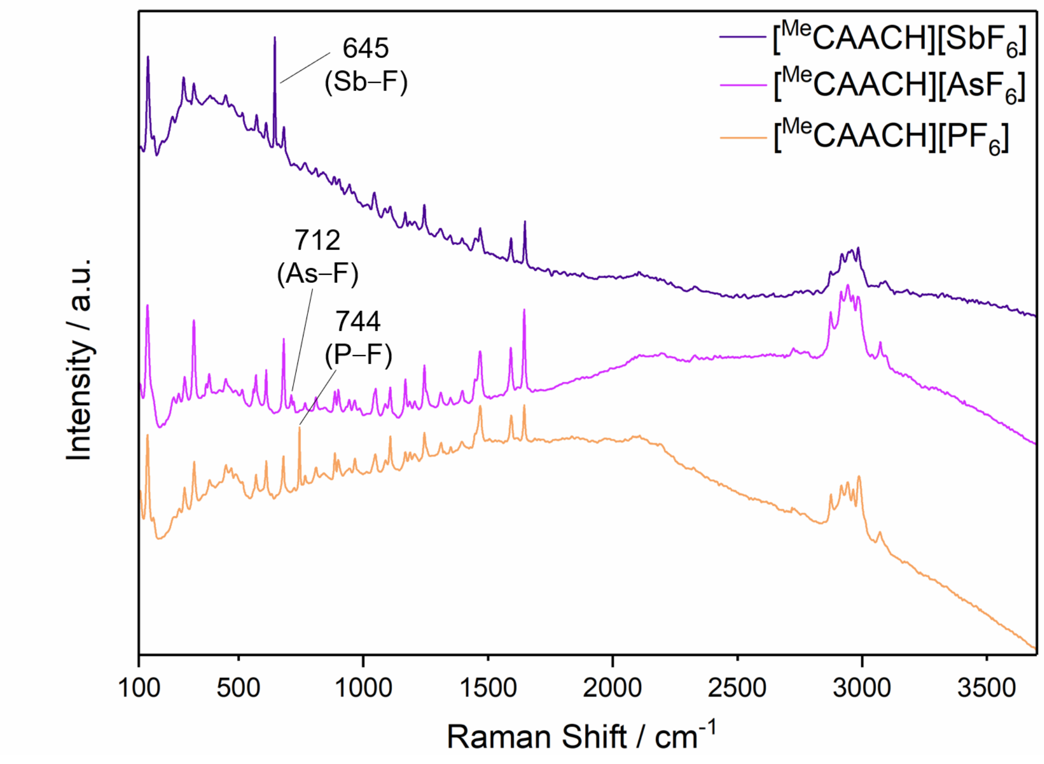

Figure S29. Raman spectra of [[Me]CAACH][PF_6], [[Me]CAACH][AsF_6] and [[Me]CAACH][SbF_6].

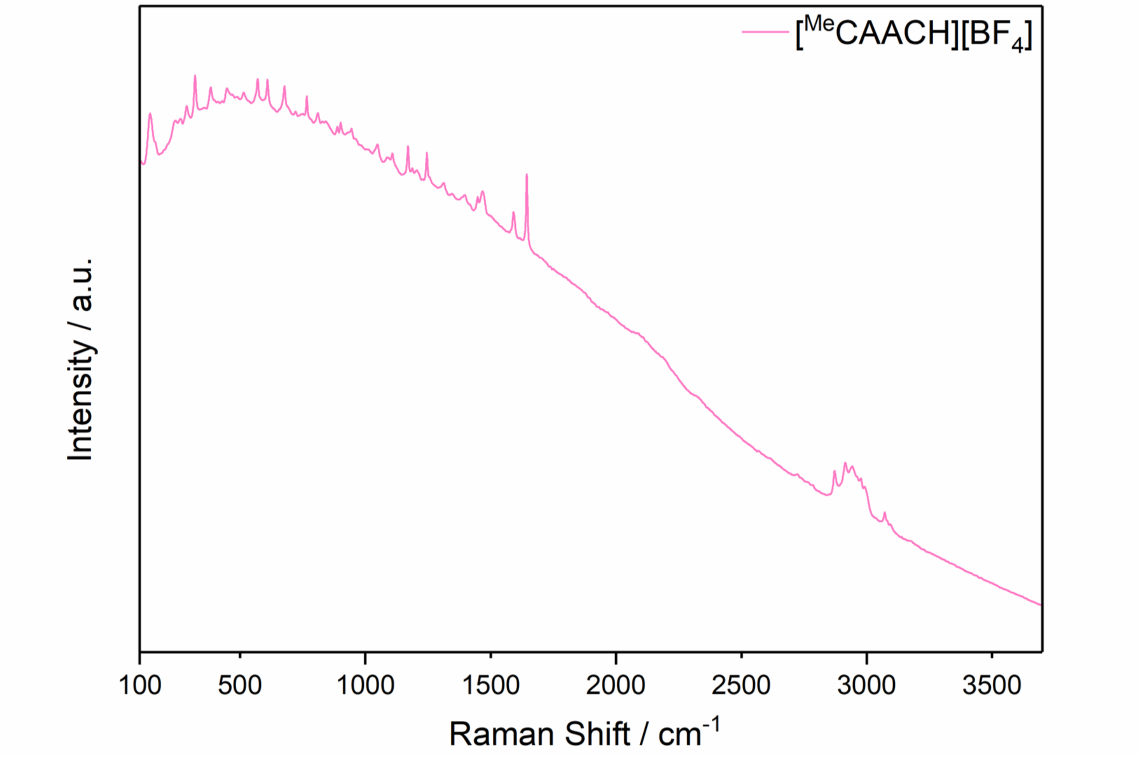

Figure S30. Raman spectra of [^{Me}CAACH][BF_4].

S3 Crystal Structure Data

Table S1. Selected crystal data for [^{Me}CAACH][SiF_5], [^{Me}CAACH][GeF_5] and [^{Me}CAACH][(THF)SnF_5].

	[^{Me}CAACH][SiF_5]	[^{Me}CAACH][GeF_5]	[^{Me}CAACH] [(THF)SnF_5]
CCDC No.	2286835	2286836	2286837
Chemical formula	$C_{20}H_{32}N{\cdot}SiF_5$	$C_{20}H_{32}N{\cdot}GeF_5$	$C_{20}H_{32}N{\cdot}SnF_5C_4H_8O$
F_W (g/mol)	409.55	454.05	572.26
T (K)	150	150	150
λ (Å)	1.54184	1.54184	1.54184
Cristal size (mm)	0.37 × 0.22 × 0.11	0.65 × 0.17 × 0.16	0.34 × 0.19 × 0.10
Crystal system	Monoclinic	Monoclinic	Monoclinic
Space group	$P2_1/n$	$P2_1/n$	$C2/c$
a (Å)	10.7357(4)	10.8904(1)	33.5653(5)
b (Å)	19.1191(7)	19.2640(2)	9.1824(1)
c (Å)	10.8661(3)	10.9361(2)	16.9348(2)
α (°)	90	90	90
β (°)	95.235(3)	95.391(1)	91.933(1)
γ (°)	90	90	90
V (Å^3)	2221.1(1)	2284.18(5)	5216.5(1)
Z	4	4	8
ρ_{calc} (g/cm^3)	1.225	1.320	1.457
μ (mm^{-1})	1.342	2.219	8.243
F(000)	872	944	2352
Θ range (°)	4.1–72.3	4.0–72.3	5.0–72.3
Index ranges	$-13 \le h \le 13$<br>$-23 \le k \le 23$<br>$-13 \le l \le 12$	$-13 \le h \le 13$<br>$-23 \le k \le 23$<br>$-13 \le l \le 13$	$-32 \le h \le 39$<br>$-11 \le k \le 11$<br>$-20 \le l \le 20$
Reflections collected	35176	62624	27893
Independent reflections	4372	4493	5085
Reflections with ($I > 2\sigma(I)$)	3724	3837	4369
R_{int}	0.0362	0.0365	0.0531
Data / restrains / parameters	4372 / 0 / 298	4493 / 0 / 298	5085 / 0 / 297
S[a]	1.076	1.072	1.060
R_1[b], wR_2[c] ($I > 2\sigma(I)$)	0.0486, 0.1351	0.0425, 0.1183	0.0338, 0.0805
R_1[b], wR_2[c] (all data)	0.0566, 0.1416	0.0489, 0.1243	0.0425, 0.0866
$\Delta\rho_{min}$, $\Delta\rho_{max}$ (eÅ^{-3})	−0.348, 0.399	−0.495, 0.462	−0.526, 1.178

[a] $S = [\Sigma(w(F_o^2 - F_c^2)^2)/(N_o - N_p)]^{1/2}$.

[b] $R_1 = ||F_o| - |F_c||/\Sigma|F_o|$.

[c] $wR_2 = [\Sigma(w(F_o^2 - F_c^2)^2)/\Sigma(w(F_o^2)^2)]^{1/2}$.

Table S2. Selected crystal data for [^{Me}CAACH][(dioxane)SnF_5]·dioxane, [^{Me}CAACH][PF_6] and [^{Me}CAACH][AsF_6].

	[^{Me}CAACH] [(dioxane)SnF_5] ·dioxane	[^{Me}CAACH][PF_6]	[^{Me}CAACH][AsF_6]
CCDC No.	2286838	2286839	2286840
Chemical formula	$C_{20}H_{32}N$· $SnF_5C_4H_8O_2$· $C_4H_8O_2$	$C_{20}H_{32}N·PF_6$	$C_{20}H_{32}N·AsF_6$
F_W (g/mol)	676.36	431.43	475.38
T (K)	150	150	150
λ (Å)	0.71073	1.54184	1.54184
Cristal size (mm)	0.83 × 0.40 × 0.26	0.42 × 0.27 × 0.17	0.36 × 0.28 × 0.25
Crystal system	Triclinic	Orthorhombic	Orthorhombic
Space group	*P*−1	*Pbca*	*Pbca*
a (Å)	11.1300(7)	15.3045(2)	15.5001(2)
b (Å)	11.7060(6)	14.9307(2)	14.9287(2)
c (Å)	13.3771(9)	19.9003(3)	20.0389(2)
α (°)	74.625(5)	90	90
β (°)	68.223(6)	90	90
γ (°)	82.213(5)	90	90
V (Å^3)	1559.2(2)	4547.4(1)	4636.9(1)
Z	2	8	8
ρ_{calc} (g/cm^3)	1.441	1.260	1.362
μ (mm^{-1})	0.881	1.559	2.462
F(000)	700	1824	1968
Θ range (°)	3.0−28.8	3.6−72.5	2.8−72.3
Index ranges	$-14 \le h \le 15$ $-15 \le k \le 15$ $-17 \le l \le 17$	$-12 \le h \le 18$ $-16 \le k \le 18$ $-24 \le l \le 17$	$-19 \le h \le 18$ $-18 \le k \le 12$ $-24 \le l \le 23$
Reflections collected	17737	17688	36394
Independent reflections	6662	4414	4573
Reflections with ($I > 2\sigma(I)$)	5610	3964	3807
R_{int}	0.0429	0.0200	0.0360
Data / restrains / parameters	6662 / 0 / 360	4414 / 0 / 261	4573 / 0 / 261
S[a]	1.066	1.050	1.044
R_1[b], wR_2[c] ($I > 2\sigma(I)$)	0.0388, 0.0865	0.0406, 0.1125	0.0350, 0.0958
R_1[b], wR_2[c] (all data)	0.0522, 0.0962	0.0448, 0.1169	0.0431, 0.1035
$\Delta\rho_{min}$, $\Delta\rho_{max}$ (eÅ^{-3})	−0.979, 0.720	−0.372, 0.319	−0.573, 0.371

[a] $S = [\Sigma(w(F_o^2 - F_c^2)^2)/(N_o - N_p)]^{1/2}$.

[b] $R_1 = ||F_o| - |F_c||/\Sigma|F_o|$.

[c] $wR_2 = [\Sigma(w(F_o^2 - F_c^2)^2)/\Sigma(w(F_o^2)^2)]^{1/2}$.

Table S3. Selected crystal data for [^{Me}CAACH][BF_4], [^{Me}CAACH][Cl] and [^{Me}CAACH][OTf].

	[^{Me}CAACH][BF_4]	[^{Me}CAACH][Cl]	[^{Me}CAACH][OTf]
CCDC No.	2286841	2286842	2286843
Chemical formula	$C_{20}H_{32}N{\cdot}BF_4$	$C_{20}H_{32}N{\cdot}Cl$	$C_{20}H_{32}N{\cdot}CF_3SO_3$
F_W (g/mol)	373.27	321.91	435.53
T (K)	150	150	150
λ (Å)	1.54184	1.54184	1.54184
Cristal size (mm)	0.57 × 0.25 × 0.20	0.40 × 0.13 × 0.06	0.51 × 0.37 × 0.15
Crystal system	Monoclinic	Monoclinic	Monoclinic
Space group	$P2_1/n$	$P2_1/c$	$P2_1/n$
a (Å)	10.2499(3)	9.4602(3)	10.6790(1)
b (Å)	19.4915(4)	9.8694(3)	16.0118(2)
c (Å)	10.6119(2)	20.8442(6)	27.0646(3)
α (°)	90	90	90
β (°)	91.377(2)	95.676(3)	97.927(1)
γ (°)	90	90	90
V (Å^3)	2119.50(8)	1936.6(1)	4583.6(1)
Z	4	4	8
ρ_{calc} (g/cm^3)	1.170	1.104	1.262
μ (mm^{-1})	0.760	1.700	1.651
F(000)	800	704	1856
Θ range (°)	4.1–72.2	4.2–72.0	3.2–72.3
Index ranges	$-12 \le h \le 11$<br>$-23 \le k \le 23$<br>$-13 \le l \le 13$	$-11 \le h \le 10$<br>$-7 \le k \le 11$<br>$-17 \le l \le 25$	$-11 \le h \le 12$<br>$-19 \le k \le 19$<br>$-28 \le l \le 33$
Reflections collected	5494	7696	36202
Independent reflections	5494	3727	8877
Reflections with ($I > 2\sigma(I)$)	4471	2937	7741
R_{int}	na	0.0288	0.0353
Data / restrains / parameters	5494 / 24 / 295	3727 / 0 / 207	8877 / 0 / 539
S[a]	1.067	1.019	1.106
R_1[b], wR_2[c] ($I > 2\sigma(I)$)	0.0571, 0.1562	0.0466, 0.1128	0.0477, 0.1194
R_1[b], wR_2[c] (all data)	0.0696, 0.1671	0.0635, 0.1244	0.0550, 0.1238
$\Delta\rho_{min}$, $\Delta\rho_{max}$ (eÅ^{-3})	−0.179, 0.320	−0.191, 0.535	−0.327, 0.413

[a] $S = [\Sigma(w(F_o^2 - F_c^2)^2)/(N_o - N_p)]^{1/2}$.

[b] $R_1 = ||F_o| - |F_c||/\Sigma|F_o|$.

[c] $wR_2 = [\Sigma(w(F_o^2 - F_c^2)^2)/\Sigma(w(F_o^2)^2)]^{1/2}$.

Table S4. Selected bond lengths (Å) and bond angles (°) for [^{Me}CAACH][SiF_5], [^{Me}CAACH][GeF_5] and [^{Me}CAACH][(THF)SnF_5].

[^{Me}CAACH][SiF_5]					
Bond lengths					
Si1–F1A	1.641(6)	Si1–F3A	1.571(4)	Si1–F5A	1.593(5)
Si1–F1B	1.64(2)	Si1–F3B	1.63(1)	Si1–F5B	1.55(2)
Si1–F2A	1.639(7)	Si1–F4A	1.619(4)	N1–C2	1.272(2)
Si1–F2B	1.60(2)	Si1–F4B	1.41(2)	C2–C3	1.486(3)
Bond angles					
F2A–Si1–F1A	176.6(6)	F3B–Si1–F1B	111(2)	F5A–Si1–F1A	88.8(3)
F2B–Si1–F1B	160(2)	F4A–Si1–F1A	93.5(4)	F5A–Si1–F2A	92.1(4)
F2B–Si1–F3B	89(1)	F4A–Si1–F2A	88.8(3)	F5A–Si1–F4A	123.5(3)
F3A–Si1–F1A	84.2(6)	F4B–Si1–F1B	78(2)	F5B–Si1–F1B	86(1)
F3A–Si1–F2A	92.5(4)	F4B–Si1–F2B	95(1)	F5B–Si1–F2B	84(1)
F3A–Si1–F4A	115.0(3)	F4B–Si1–F3B	121(1)	F5B–Si1–F3B	111(1)
F3A–Si1–F5A	121.3(3)	F4B–Si1–F5B	128(2)	N1–C2–C3	114.8(2)
[^{Me}CAACH][GeF_5]					
Bond lengths					
Ge1–F1A	1.734(9)	Ge1–F3A	1.697(4)	Ge1–F5A	1.73(1)
Ge1–F1B	1.68(1)	Ge1–F3B	1.67(1)	Ge1–F5B	1.63(2)
Ge1–F2A	1.731(7)	Ge1–F4A	1.651(5)	N1–C2	1.267(3)
Ge1–F2B	1.68(1)	Ge1–F4B	1.80(1)	C2–C3	1.489(3)
Bond angles					
F1B–Ge1–F4B	92(1)	F3B–Ge1–F1B	87.8(8)	F5A–Ge1–F1A	87.3(5)
F2A–Ge1–F1A	174.3(5)	F3B–Ge1–F2B	92(1)	F5A–Ge1–F2A	91.1(5)
F2B–Ge1–F1B	176(1)	F3B–Ge1–F4B	104(1)	F5B–Ge1–F1B	93(1)
F2B–Ge1–F4B	92.4(8)	F4A–Ge1–F1A	89.9(5)	F5B–Ge1–F2B	85(1)
F3A–Ge1–F1A	88.3(5)	F4A–Ge1–F2A	95.6(5)	F5B–Ge1–F3B	145(1)
F3A–Ge1–F2A	88.3(3)	F4A–Ge1–F3A	114.7(3)	F5B–Ge1–F4B	110(1)
F3A–Ge1–F5A	126.0(5)	F4A–Ge1–F5A	119.0(5)	N1–C2–C3	115.1(2)
[^{Me}CAACH][(THF)SnF_5]					
Bond lengths					
Sn1–F1	1.945(2)	Sn1–F5	1.952(2)	C3–C4	1.511(6)
Sn1–O1	2.167(2)	O1–C1	1.452(4)	N11–C12	1.280(4)
Sn1–F2	1.937(2)	O1–C4	1.461(4)	C12–C13	1.485(4)
Sn1–F3	1.945(2)	C1–C2	1.501(6)		
Sn1–F4	1.940(2)	C2–C3	1.518(7)		
Bond angles					
F1–Sn1–O1	177.61(9)	F3–Sn1–O1	86.0(1)	C1–O1–C4	110.8(3)
F1–Sn1–F3	92.7(1)	F3–Sn1–F5	171.87(9)	C4–O1–Sn1	125.3(2)
F1–Sn1–F5	95.38(9)	F4–Sn1–F1	96.0(1)	O1–C1–C2	104.5(3)
F2–Sn1–F1	93.08(9)	F4–Sn1–O1	86.1(1)	C1–C2–C3	103.0(4)
F2–Sn1–O1	84.86(9)	F4–Sn1–F3	90.4(1)	C4–C3–C2	102.7(3)
F2–Sn1–F3	89.1(1)	F4–Sn1–F5	88.2(1)	O1–C4–C3	103.8(3)
F2–Sn1–F4	170.90(9)	F5–Sn1–O1	85.88(9)	N11–C12–C13	114.3(3)
F2–Sn1–F5	91.06(9)	C1–O1–Sn1	123.3(2)		

Table S5. Selected bond lengths (Å) and bond angles (°) for [^{Me}CAACH][(dioxane)SnF_5]·dioxane, [^{Me}CAACH][PF_6] and [^{Me}CAACH][AsF_6].

[^{Me}CAACH][(dioxane)SnF_5]·dioxane					
Bond lengths					
Sn1–F1	1.925(2)	Sn1–F5	1.941(2)	O2–C3	1.412(4)
Sn1–O1	2.177(2)	O1–C1	1.444(4)	C3–C4	1.483(5)
Sn1–F2	1.926(2)	O1–C4	1.458(4)	N11–C12	1.276(4)
Sn1–F3	1.931(2)	C1–C2	1.477(6)	C12–C13	1.482(4)
Sn1–F4	1.935(2)	O2–C2	1.426(4)		
Bond angles					
F1–Sn1–O1	85.64(9)	F2–Sn1–F5	172.90(9)	C1–O1–C4	109.6(3)
F1–Sn1–F2	90.9(1)	F3–Sn1–O1	178.62(9)	C4–O1–Sn1	123.3(2)
F1–Sn1–F3	94.48(9)	F3–Sn1–F4	95.93(8)	O1–C1–C2	108.4(3)
F1–Sn1–F4	169.59(8)	F3–Sn1–F5	94.84(9)	C3–O2–C2	108.9(3)
F1–Sn1–F5	88.98(9)	F4–Sn1–O1	83.96(8)	O2–C2–C1	111.2(4)
F2–Sn1–O1	86.4(1)	F4–Sn1–F5	90.00(8)	O2–C3–C4	111.4(3)
F2–Sn1–F3	92.25(9)	F5–Sn1–O1	86.5(1)	O1–C4–C3	109.0(3)
F2–Sn1–F4	88.87(9)	C1–O1–Sn1	124.2(2)	N11–C12–C13	114.5(3)
[^{Me}CAACH][PF_6]					
Bond lengths					
P1–F1	1.585(1)	P1–F4	1.597(1)	N1–C2	1.274(2)
P1–F2	1.600(1)	P1–F5	1.588(1)	C2–C3	1.490(2)
P1–F3	1.583(1)	P1–F6	1.594(1)		
Bond angles					
F1–P1–F2	179.02(8)	F3–P1–F4	88.81(6)	F5–P1–F6	89.49(8)
F1–P1–F4	90.24(7)	F3–P1–F5	178.92(8)	F6–P1–F2	89.67(7)
F1–P1–F5	90.24(8)	F3–P1–F6	91.38(8)	F6–P1–F4	178.99(7)
F1–P1–F6	90.75(7)	F4–P1–F2	89.34(6)	N1–C2–C3	114.7(1)
F3–P1–F1	90.38(8)	F5–P1–F2	88.88(8)		
F3–P1–F2	90.49(8)	F5–P1–F4	90.30(7)		
[^{Me}CAACH][AsF_6]					
Bond lengths					
As1–F1	1.703(2)	As1–F4	1.708(2)	N1–C2	1.273(3)
As1–F2	1.708(2)	As1–F5	1.714(2)	C2–C3	1.491(3)
As1–F3	1.708(2)	As1–F6	1.719(2)		
Bond angles					
F1–As1–F2	89.8(1)	F2–As1–F4	90.4(1)	F4–As1–F5	90.10(9)
F1–As1–F3	91.8(1)	F2–As1–F5	90.32(9)	F4–As1–F6	89.15(9)
F1–As1–F4	178.58(9)	F2–As1–F6	179.6(1)	F5–As1–F6	89.53(8)
F1–As1–F5	88.50(8)	F3–As1–F4	89.6(1)	N1–C2–C3	114.7(2)
F1–As1–F6	90.6(1)	F3–As1–F5	178.97(8)		
F2–As1–F3	90.66(9)	F3–As1–F6	89.49(8)		

Table S6. Selected bond lengths (Å) and bond angles (°) for [^{Me}CAACH][BF_4], [^{Me}CAACH][Cl] and [^{Me}CAACH][OTf].

[^{Me}CAACH][BF_4]					
Bond lengths					
B1–F1A	1.405(6)	B1–F3A	1.344(4)	N1–C2	1.272(3)
B1–F1B	1.33(2)	B1–F3B	1.26(2)	C2–C3	1.487(3)
B1–F2A	1.337(6)	B1–F4A	1.385(5)		
B1–F2B	1.32(1)	B1–F4B	1.27(2)		
Bond angles					
F1A–B1–F2A	107.5(4)	F1B–B1–F4B	115(1)	F3A–B1–F4A	106.4(3)
F1B–B1–F2B	112(1)	F2A–B1–F3A	112.5(5)	F3B–B1–F4B	117(2)
F1A–B1–F3A	110.3(4)	F2B–B1–F3B	100(1)	N1–C2–C3	114.9(2)
F1B–B1–F3B	105(1)	F2A–B1–F4A	111.6(4)		
F1A–B1–F4A	108.5(3)	F2B–B1–F4B	107(1)		
[^{Me}CAACH][Cl]					
Bond lengths				*Bond angles*	
N1–C2	1.276(2)	C2–C3	1.489(3)	N1–C2–C3	113.8(2)
[^{Me}CAACH][OTf]					
Ion Pair 1					
Bond lengths					
S1–O1	1.438(2)	S1–C22	1.830(2)	C22–F3	1.334(3)
S1–O2	1.431(2)	C22–F1	1.317(3)	N1–C2	1.274(3)
S1–O3	1.434(2)	C22–F2	1.325(3)	C2–C3	1.488(3)
Bond angles					
O1–S1–C22	103.0(1)	O3–S1–C22	103.6(1)	F2–C22–F3	107.1(2)
O2–S1–O1	115.2(1)	F1–C22–S1	112.4(2)	F3–C22–S1	110.9(2)
O2–S1–O3	115.3(1)	F1–C22–F2	107.3(2)	N1–C2–C3	114.7(2)
O2–S1–C22	102.6(1)	F1–C22–F3	107.4(2)		
O3–S1–O1	114.6(1)	F2–C22–S1	111.5(2)		
Ion Pair 2					
Bond lengths					
S2–O4	1.432(2)	S2–C52	1.826(2)	C52–F6	1.328(3)
S2–O5	1.437(2)	C52–F4	1.325(3)	N31–C32	1.274(3)
S2–O6	1.440(2)	C52–F5	1.327(3)	C32–C33	1.492(3)
Bond angles					
O4–S2–O5	115.1(1)	O6–S2–C52	102.9(1)	F5–C52–F6	106.8(2)
O4–S2–O6	115.7(1)	F4–C52–S2	110.7(2)	F6–C52–S2	111.5(2)
O4–S2–C52	103.5(1)	F4–C52–F5	107.3(2)	N31–C32–C33	114.3(2)
O5–S2–O6	117.0(1)	F4–C52–F6	108.6(2)		
O5–S2–C52	103.2(1)	F5–C52–S2	111.7(2)		

S3.1 Crystal structures of selected compounds

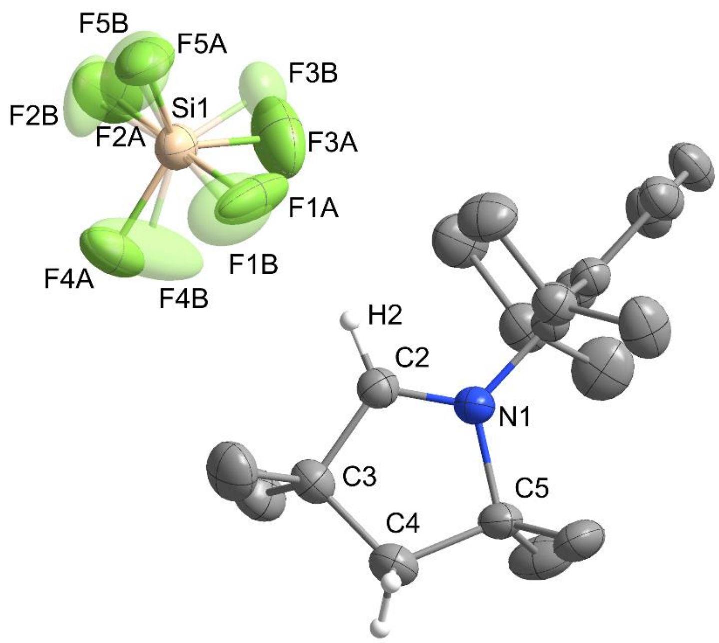

(a)

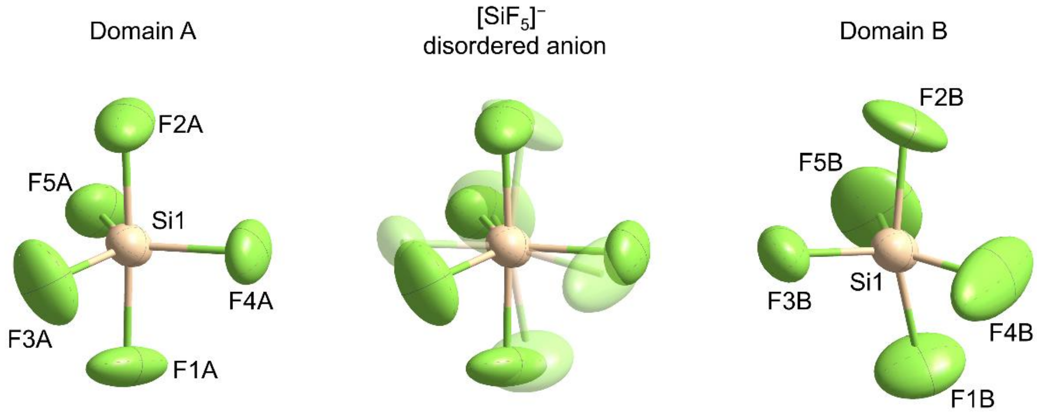

(**b**)

Figure S31. Structure of the disordered [^{Me}CAACH][SiF_5]: **(a)** asymmetric unit of [^{Me}CAACH][SiF_5] with the disordered anion; and **(b)** structure of the disordered anion. The ellipsoids are drawn at 50% probability. The positions of disordered atoms are shown in domains A and B. For clarity, all hydrogen atoms are omitted and doman B is shaded, when domain A and B are overlaping. The occupancies of domain A and B are 75% and 25%, respectively.

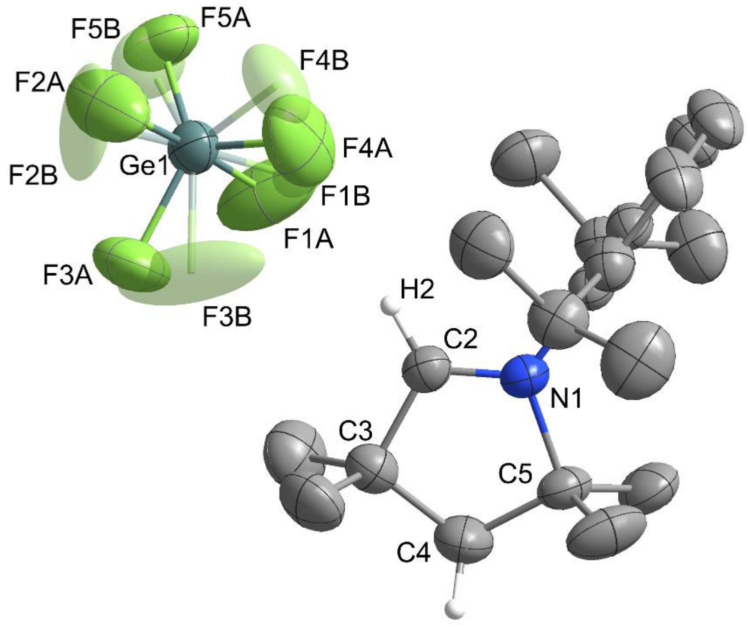

(a)

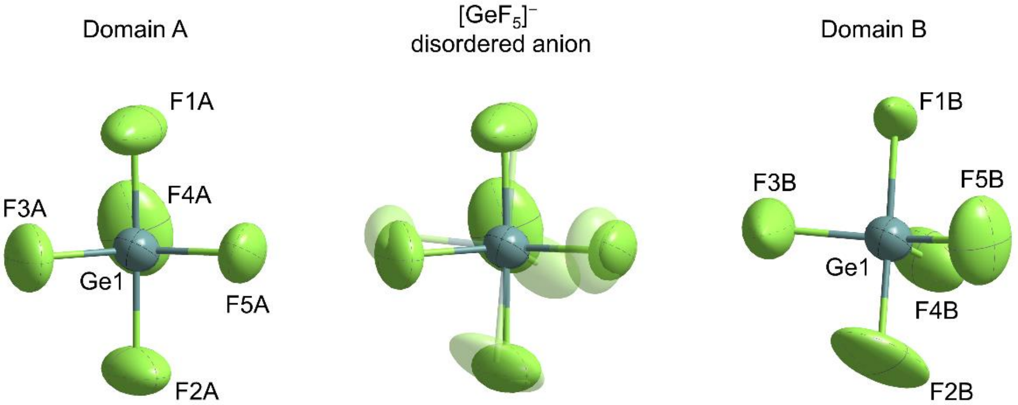

(**b**)

Figure S32. Structure of the disordered [^{Me}CAACH][GeF_5]: **(a)** asymmetric unit of [^{Me}CAACH][GeF_5] with the disordered anion; and **(b)** structure of the disordered anion. The ellipsoids are drawn at 50% probability. The positions of disordered atoms are shown in domains A and B. For clarity, all hydrogen atoms are omitted and doman B is shaded, when domain A and B are overlaping. The occupancies of domain A and B are 67% and 33%, respectively.

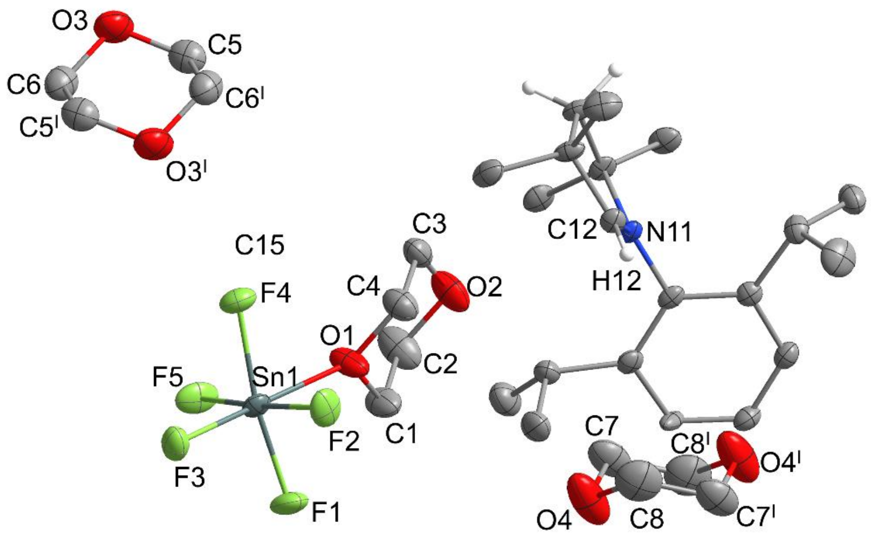

Figure S33. Crystal structure of [^{Me}CAACH][(dioxane)SnF_5]·dioxane with solvent molecules. The ellipsoids are drawn at 50% probability. For clarity, all hydrogen atoms are omitted except those on the heterocyclic ring.

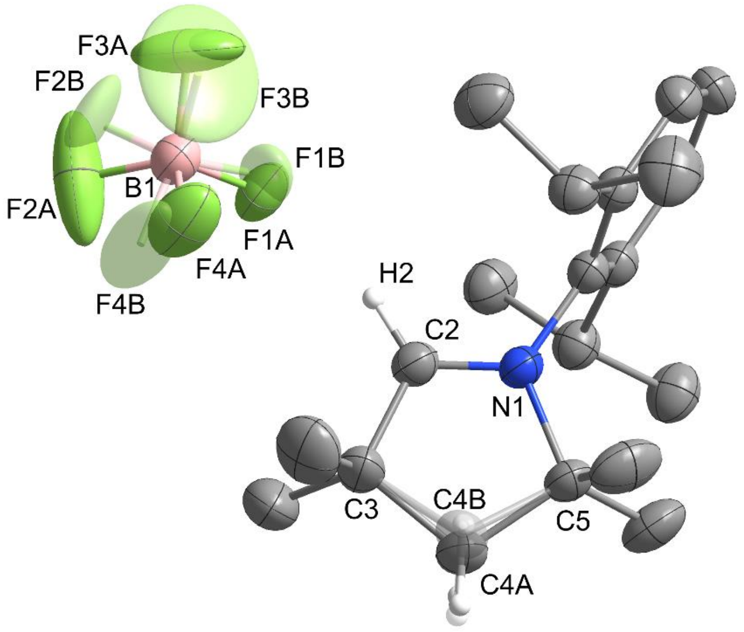

(a)

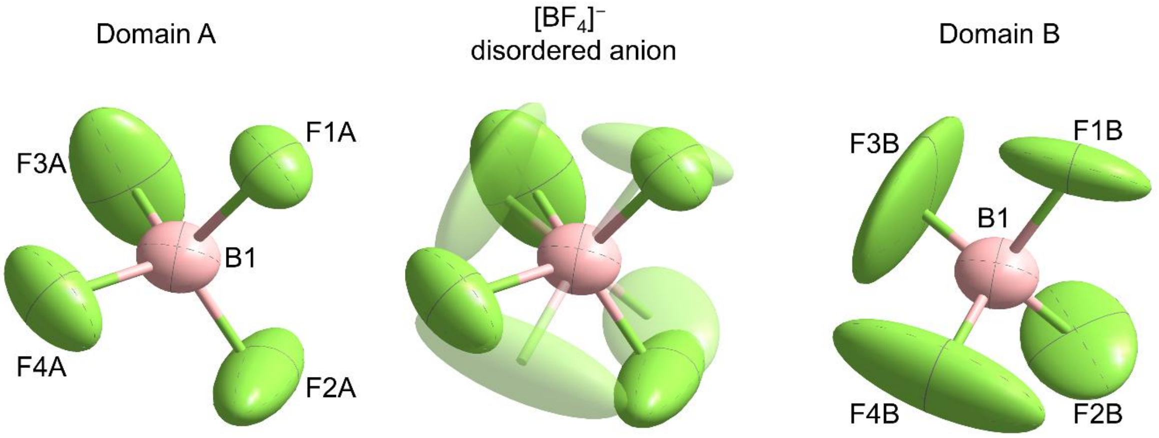

(b)

Figure S34. Structure of the disordered [^{Me}CAACH][BF_4]: (a) asymmetric unit of [^{Me}CAACH][BF_4] with the disordered anion; and (b) structure of the disordered anion. The ellipsoids are drawn at 50% probability. The positions of disordered atoms are shown in domains A and B. For clarity, all hydrogen atoms are omitted and doman B is shaded, when domain A and B are overlaping. The occupancies of domain A and B are 73% and 27%, respectively.

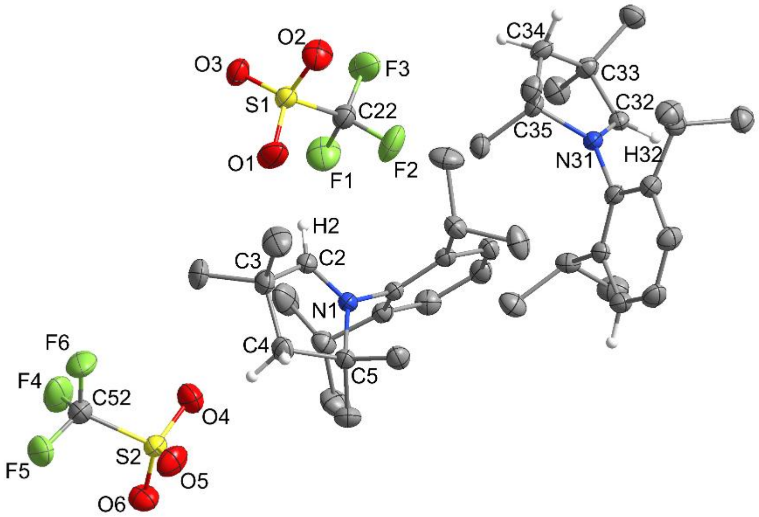

Figure S35. Structure of the asymmetric unit of [^{Me}CAACH][OTf]. The ellipsoids are drawn at 50% probability. For clarity, all hydrogen atoms are omitted except those on the heterocyclic ring.
